# Identification of *TIMELESS* and *RORA* as key clock molecules of non-small cell lung cancer and the comprehensive analysis

**DOI:** 10.1186/s12885-022-09203-1

**Published:** 2022-01-25

**Authors:** Haocheng Xian, Yuan Li, Boliang Zou, Yajuan Chen, Houqing Yin, Xuejun Li, Yan Pan

**Affiliations:** 1 Department of Pharmacology, School of Basic Medical Sciences, Health Science Center, Peking University, Beijing, 100191 China; 2 Department of Rehabilitation Medicine, Kunming Medical University, 1168 Western Chunrong Road, Yuhua Street, Chenggong District, Kunming, 650500 Yunnan China; 3 Beijing Key Laboratory of Tumor Systems Biology, Peking University, Beijing, 100191 China

**Keywords:** Non-small cell lung cancer, Circadian clock genes, *TIMELESS*, *RORA*, Bioinformatics

## Abstract

**Background:**

The incidence rate of non-small cell lung cancer (NSCLC) has been increasing worldwide, and the correlation of circadian rhythm disruption with a raised risk of cancer and worse prognosis has been shown by accumulating evidences recently. On the other hand, drug resistance and the impact of tumor heterogeneity have been inevitable in NSCLC therapy. These both lead to an urgent need to identify more useful prognostic and predictive markers for NSCLC diagnosis and treatment, especially on the aspect of circadian clock genes.

**Methods:**

The expression of the main clock genes in cancer was probed with TIMER and Oncomine databases. The prognostic value of key clock genes was probed systematically with the Kaplan–Meier estimate and Cox regression on samples from TCGA database. RT-qPCR was performed on patient tissue samples to further validate the results from databases. The functional enrichment analysis was performed using the “ClusterProfiler” R package, and the correlation of key clock genes with tumor mutation burden, immune checkpoint, and immune infiltration levels were also assessed using multiple algorithms including TIDE, TIMER2.0, and XCELL.

**Results:**

*TIMELESS* was significantly upregulated in lung tissue of clinical lung cancer patients as well as TCGA and Oncomine databases, while *RORA* was downregulated. Multivariate Cox regression analysis indicated that *TIMELESS* (*P* = 0.004, HR = 1.21 [1.06, 1.38]) and *RORA* (*P* = 0.047, HR = 0.868 [0.755, 0.998]) has a significant correlation with overall survival in NSCLC. Genes related to *TIMELESS* were enriched in the cell cycle and immune system, and the function of *RORA* was mainly focused on oncogenic signaling pathways or glycosylation and protein activation. Also, *TIMELESS* was positively correlated with tumor mutation burden while *RORA* was negatively correlated with it. *TIMELESS* and *RORA* were also significantly correlated with immune checkpoint and immune infiltration levels in NSCLC. Additionally, *TIMELESS* showed a significant positive relationship with lipid metabolism.

**Conclusions:**

*TIMELESS* and *RORA* were identified as key clock genes in NSCLC, and were independent prognostic factors for overall survival in NSCLC. The function of them were assessed in many aspects, indicating the strong potential of the two genes to serve as biomarkers for NSCLC progression and prognosis.

**Supplementary Information:**

The online version contains supplementary material available at 10.1186/s12885-022-09203-1.

## Introduction

As the most common cancer type, lung cancer caused most cancer-related deaths worldwide, accounting for about 1.4 million deaths per year [1]. According to the cell origin, there are small-cell lung cancer and non-small cell lung cancer (NSCLC), in which 85% are NSCLC [2]. Although considerable efforts have been paid to elucidate its development and progression, the molecular mechanisms underlying NSCLC remain unclear [3]. Recently, compared with traditional chemotherapy, precision medicine, targeted therapy, and immunotherapy have led to an improvement of treatment outcomes in patients with NSCLC. However, drug resistance and the impact of tumor heterogeneity have been inevitable [4]. Therefore, the need to identify more useful prognostic and predictive markers or targets for the diagnosis and treatment of NSCLC is more and more urgent.

The circadian rhythm refers to the physiological and behavioral oscillations of an organism in a cycle of about 24 h, behind which is a complicated system. The occurrence of circadian rhythm in mammals is based on endogenous clock genes, which mainly consist of Clock circadian regulator, brain and muscle ARNT-like 1, period circadian regulator 1 (*PER1*), *PER2*, cryptochrome circadian regulator 1 (*CRY1*), *CRY2*, timeless circadian regulator (*TIMELESS*), retinoic acid-related orphan receptor A (*RORA*), nuclear receptor subfamily 1, group D member 1 (*NR1D1*), and *NR1D2* [5]. They synchronize multiple molecular, biochemical, physiological, and behavioral process, also, at the cellular level, lots of genes were regulated in expression or function transcriptionally or post-transcriptionally by these clock genes [6].

Circadian disruption is one of the group 2A human carcinogens identified by WHO [7]. Many epidemiological studies have shown that disorder of the circadian rhythm is related to worse prognosis and increased risks of cancer. For example, long-period shiftwork is correlated with higher risk of the development of prostate and breast cancers [8]. Also, the hazard ratio of rectal cancer [9] and lung cancer [10] is increased after exposure to shiftwork. Some in vivo experiments have also confirmed the pattern: hypothalamic suprachiasmatic nucleus ablation in normal mice or tumor-bearing mice strongly accelerates tumor progression [11, 12]. In addition, mice with genetic alterations in some clock genes can more easily develop teratomas, lymphoma, liver cancer, and ovarian cancer [13–15]. However, to date, the detailed effects of clock genes on NSCLC as well as their underlying mechanisms remain unclear.

As technology has developed, bioinformatics has been widely applied to genomic and proteomic studies and has provided an unparalleled scale to explore the correlation and interaction of molecules and many biological processes. In this study, using bioinformatics analysis, we identified *TIMELESS* and *RORA* as the key clock genes in NSCLC and showed their strong potential as key molecules underlying NSCLC progression and prognosis.

## Materials and methods

### Datasets and data availability

The sex, age, survival, status, topography and tumor-node-metastasis (TNM) stage of lung adenocarcinoma (LUAD) and lung squamous cell carcinoma (LUSC) samples were extracted from The Cancer Genome Atlas (TCGA: https://cancergenome.nih.gov) with the exclusion patients without sufficient data were excluded from subsequent analyses. In total, 513 TCGA-LUAD samples and 501 TCGA-LUSC samples were qualified and used for subsequent analyses. Data from normal lung tissue samples were also obtained in Genotype-Tissue Expression (GTEx) (https://gtexportal.org/home/datasets).

### Clinical patient tissue sample collection

After the approval of the ethics committees of the Third Affiliated Hospital of Kunming Medical University (No: KYCS202180), paired tumor tissue and adjacent normal tissue were obtained in Yunnan Cancer Hospital from 13 patients who was diagnosed with lung cancer. Written informed consent was obtained from all patients before the tissue sample collection.

### Oncomine database analyses

As a comprehensive bioinformatics database, the Oncomine database (https://www.oncomine.org/) is established for providing and assessing cancer transcriptome data [16]. In this study, we compared the transcription levels of circadian genes between tumor specimens and normal tissue. The threshold value was set as follows: fold change more than 1.5, *P* value less than 0.001, and gene ranking in top 10%.

### Kaplan–Meier plotter database assay

The Kaplan–Meier plotter (KM-plotter, http://kmplot.com/analysis/) includes 10,461 samples of cancer patients and can probe the effects of multiple genes on survival [17]. In our study, this database was used to analyze correlation of *TIMELESS*, *RORA*, *PER1*, *PER2*, and *CRY2* expression with survival in lung cancer. The log-rank *P* value were calculated as well as the hazard ratio (HR) with 95% confidence intervals (CIs).

### HR and overall survival nomogram model

To identify the proper terms for the nomogram, we did univariate and multivariate Cox regression analyses. The *P* value, HR, and 95% CI of each variable were plotted in the forest plot. And a nomogram model based on the multivariate Cox regression model was developed as a visual representation to predict the recurrence risk of 1, 2, 3, and 5 years, respectively. “Forestplot” and “rms” of R software package 4.0.3 were used.

### RT-qPCR

Total RNA was extracted from clinical samples using TRIzol reagent (Invitrogen, California, USA) according to the manufacture’s protocol, and the cDNA was synthesized using the cDNA First Strand Synthesis Kit (Abclonal, WuHan, China). RT-qPCR was performed according to the instructions of SYBR Green Fast qPCR Mix (Mei5bio, Beijing, China) with following conditions: 95 °C for 30 s, then 45 cycles including 95 °C for 5 s and 60 °C for 30 s for annealing and elongation. The relative expression of target genes was normalized to GAPDH and quantified with 2^-ΔΔCt^ method [18]. The oligonucleotide primer sequences used were as follows: *TIMELESS* forward 5′- GTTTTGGCAATCTGCCTAAGGA-3′, and reverse 5′- GCAGCTCATACAAGGTTTCACT-3′; *RORA* forward 5′- CACGACGACCTCAGTAACTACA-3′, and reverse 5′- TGGTGAACGAACAGTAGGGAA-3′; *GAPDH*, forward 5′- GAGTCCACTGGCGTCTTCAC-3′, and reverse 5′- TGGTTCACACCCATGACGAA-3′.

### Identification of genes related to *TIMELESS* and *RORA* in NSCLC samples

The “Limma” R package was applied to assess the mRNA expression of samples with higher and lower *TIMELESS* and *RORA* expression divided according to the median. The threshold was defined as adjusted *P* value (FDR < 0.05) and the fold change (more than 1.5). The package version used in the present study is 3.18.0.

### Functional enrichment analyses

The “ClusterProfiler” R package was used to perform Gene Ontology (GO) and Kyoto Encyclopedia of Genes and Genomes (KEGG) pathway enrichment analyses with those whole genomic genes as enrichment background [19]. Terms with a *P*-value < 0.01 and an enrichment factor > 1.5 were grouped into clusters based on the membership similarities. The detailed method was as described previously [20].

### Analyses of tumor mutation burden and correlation with gene expression levels

The correlation of *TIMELESS* and *RORA* expression from TCGA database with tumor mutation burden were analyzed by Spearman’s correlation analysis. And the results were visualized with the “ggplot2” package (tumor mutation burden [TMB] score and genes) and “pheatmap” package (gene correlations) of R software 4.0.3. *P* < 0.05 has statistical significance.

### Prediction of immune checkpoint blockade response

The tumor immune dysfunction and exclusion (TIDE) algorithm was mainly used to predict the immune checkpoint blockade (ICB) response as previously described [21]. Multiple gene expression markers were used to estimate the dysfunction and exclusion of tumor infiltration cytotoxic T lymphocytes (CTLs).

### Analysis of immune infiltration level

As a comprehensive database, TIMER2.0 (http://timer.comp-genomics.org/) could predict and estimate the immune infiltration levels by multiple immune deconvolution methods [22]. In this study, it was used to explore the correlations of immune cell infiltration levels (CD8+ T cells, CD4+ T cells, B cells, myeloid dendritic cell [mDCs], neutrophils, and macrophages) with *TIMELESS* and *RORA* expression adjusted by tumor purity. The correlation of immune infiltration levels with *TIMELESS* and *RORA* expression levels was also analyzed by the “immuneeconv” R package. The data were visualized using the “ggplot2” package and “pheatmap” package. Spearman’s correlation analysis was used and *P* < 0.05 was considered to have statistical significance.

### The mutation situation analysis

cBioportal (cbioportal.org) is a comprehensive tool for multidimensionally assessing cancer data from the level of genomics [23]. In this study, the cBioportal database was used to examine the mutation situation of *TIMELESS* and *RORA* in NSCLC samples, and lollipop plots were plotted for each gene.

### Statistical analysis

Data are shown as the mean ± SD. The correlation was evaluated by Spearman’s correlation analysis. Survival curves were generated by the Kaplan-Meier method with the log-rank test. Student’s *t*-test was used to compare the difference within two groups, and the Kruskal-Wallis test was used to compare the difference among more than two groups unless otherwise stated. *P* < 0.05 was considered statistically significant.

## Results

### The mRNA levels of five clock genes (*TIMELESS*, *RORA*, *PER1*, *PER2*, *CRY2*) are significantly altered in several kinds of cancer

**Fig. 1:**
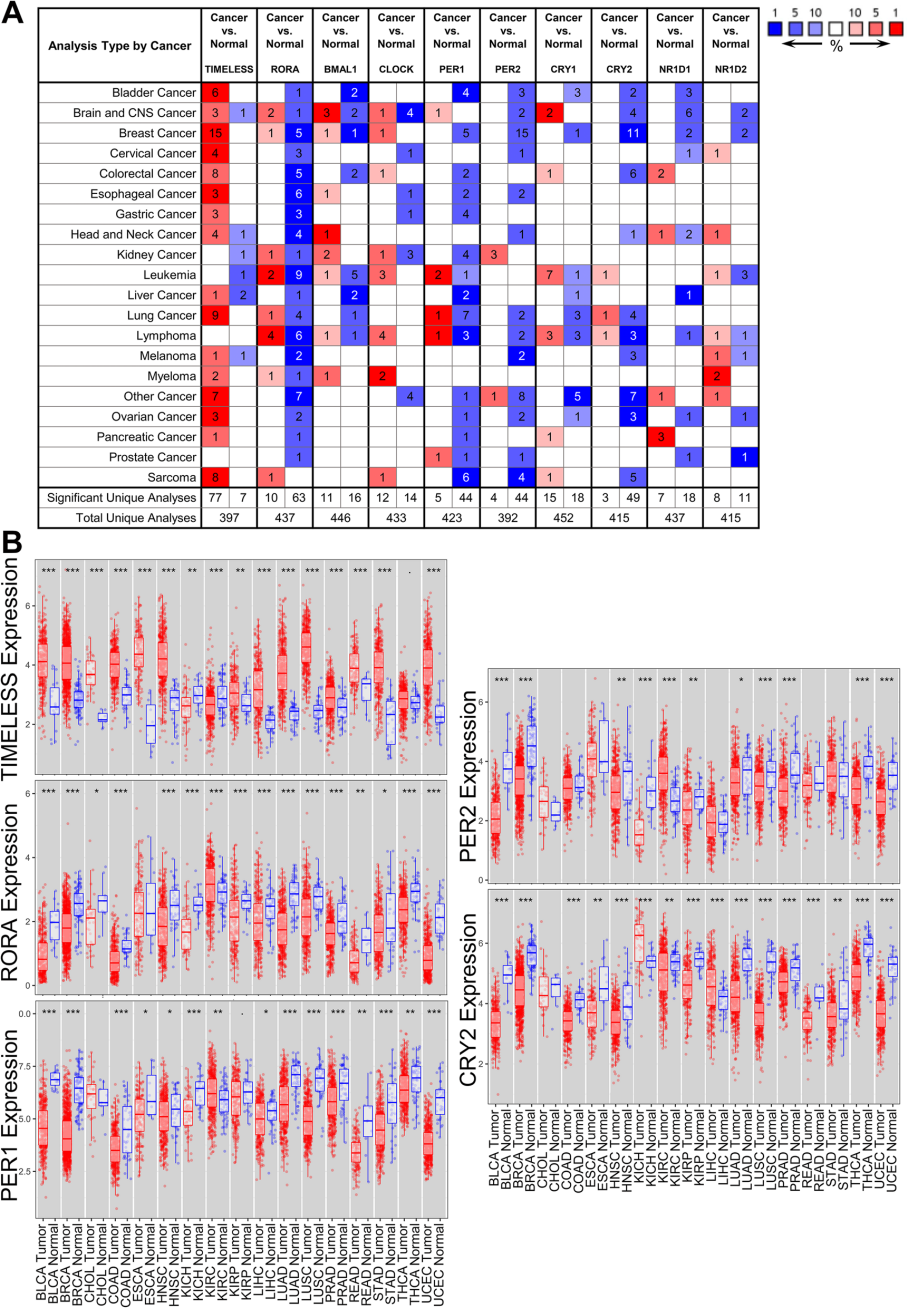
The mRNA expression level of clock genes in different cancers. **A** Datasets in which the mRNA expression levels of clock genes were significantly changed compared with normal tissues in Oncomine database. Color red represents upregulated genes while blue represents downregulated genes. The depth of the cell color represents the rank of target genes in all the significantly changed genes in a dataset. The threshold was set as: *P*-value of 0.001, fold change of 1.5, and gene ranking of top 10% **B** Expression levels of *TIMELESS*, *RORA*, *PER1*, *PER2*, and *CRY2* in different cancer types from TCGA database were analyzed by TIMER. *P < 0.05, **P < 0.01, ***P < 0.001

The transcription level of clock genes in tumor and normal tissues was assessed with Oncomine database (Fig. 1A). we found that the *TIMELESS* expression level was upregulated in most of the significantly altered datasets (77 of 84 datasets, 91.7%), especially in breast cancer and lung cancer, whereas the expression levels of *RORA*, *PER1*, *PER2*, and *CRY2* were downregulated in most of the significantly altered datasets (63 of 73 datasets, 86.3%; 44 of 49 datasets, 89.8%; 44 of 48 datasets, 91.7%; 49 of 52 datasets, 94.2%).

To further confirm the alterations in transcription level of these five clock genes, we repeated the mRNA levels detection of *TIMELESS*, *RORA*, *PER1*, *PER2*, and *CRY2* using the TCGA RNA-seq data (Fig. 1B). *TIMELESS* expression was upregulated significantly in most cancer types (*P* < 0.05), while it was downregulated in kidney cancer (KICH and KIRC). *RORA* expression was significantly lower in multiple cancer types than that in corresponding normal tissues (*P* < 0.05), while it was upregulated in KIRC.

### *TIMELESS* and *RORA* were identified as key clock genes in NSCLC

**Fig. 2:**
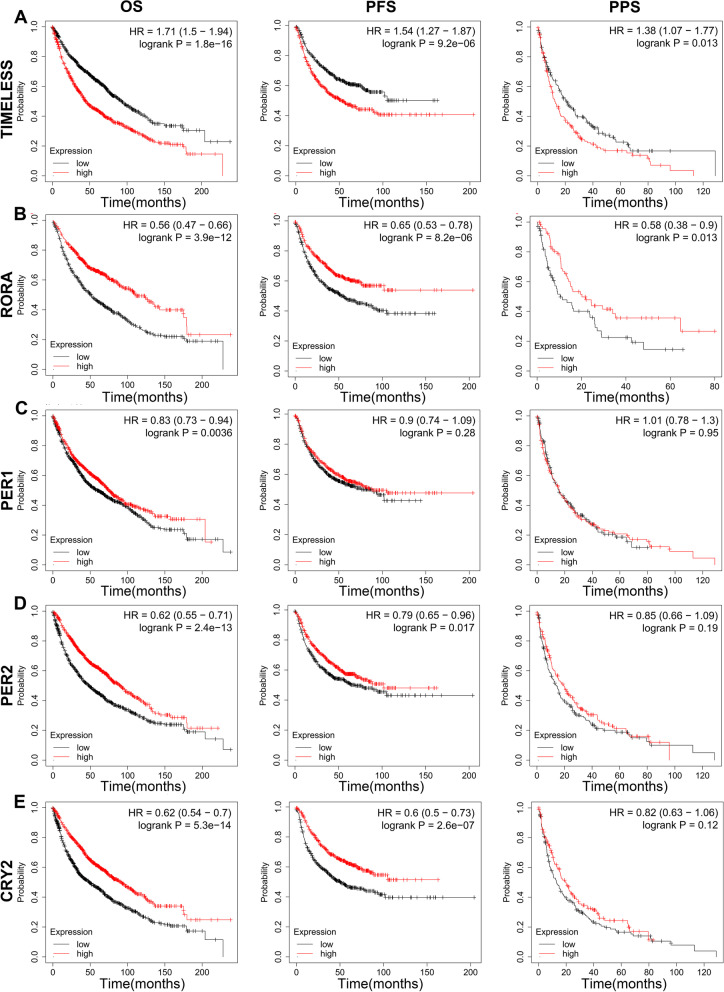
Kaplan-Meier survival curve of clock genes. The correlations of OS, PFS, and PPS with *TIMELESS* (**A**), *RORA* (**B**), *PER1* (**C**), *PER2* (**D**), and *CRY2* (**E**) expression in lung cancer were probed using KM plotter database. The hazard ratio (HR) and log-rank *P*-value were also shown on each curve

As *TIMELESS* expression was significantly upregulated and *RORA*, *PER1*, *PER2*, and *CRY2* expression was significantly downregulated in most cancer types including LUAD and LUSC, it is necessary to probe their prognostic values (Fig. 2A-E). The log-rank test indicated a significant association of mRNA levels with overall survival (OS) of lung cancer patients (*P* < 0.05), and the *TIMELESS*, *RORA*, *PER2*, and *CRY2* expressions were significantly correlated with progression-free survival (PFS) in lung cancer patients (*P* < 0.05). The *TIMELESS* and *RORA* expression levels were also significantly correlated with post-progression survival (PPS; *P* < 0.05). Collectively, lung cancer patients with high expression of *TIMELESS* or low expression of *RORA*, *PER1*, *PER2*, or *CRY2* had a significantly poorer prognosis in OS.

**Fig. 3:**
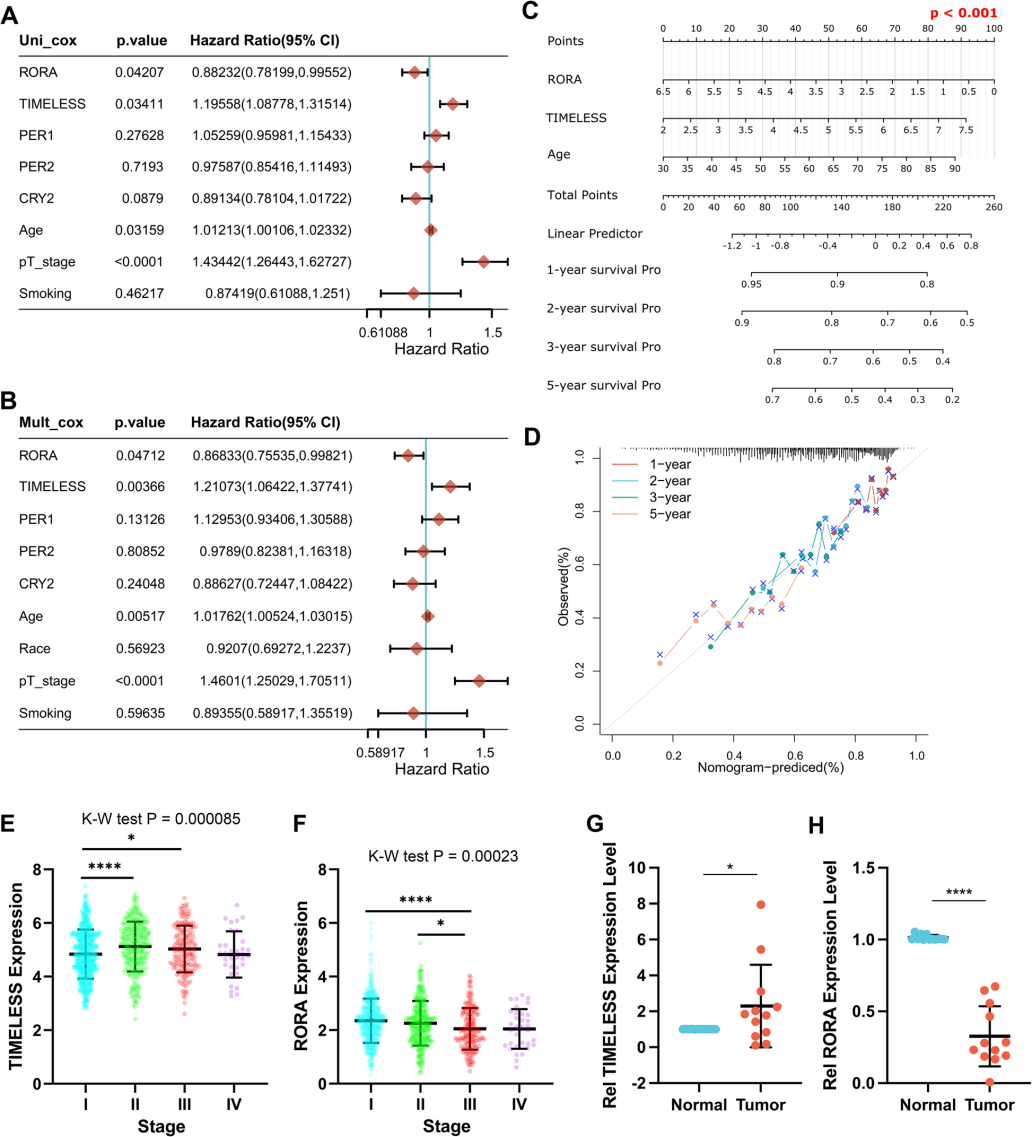
Prognostic value of *TIMELESS*, *RORA*, *PER1*, *PER2*, and *CRY2*. The univariate (**A**) and multivariate cox regression analysis (**B**) of *TIMELESS*, *RORA*, *PER1*, *PER2*, and *CRY2* levels as well as some clinical parameters including age, race, pathological tumor stage, and smoking status with overall survival in NSCLC samples from TCGA. **C** The nomogram to predict the 1,2,3, and 5-year overall survival of NSCLC patients by adding up the points of *RORA*, *TIMELESS*, age to the total points. **D** The calibration curve for the overall survival nomogram model. A dashed diagonal line represents the ideal nomogram, and four colored lines represent the 1, 2, 3, and 5-year observed nomograms. **E**-**F** Correlation of *TIMELESS* (**E**) and *RORA* (**F**) expression with pTNM stage of NSCLC in TCGA database. **G**-**H** *TIMELESS* (**G**) and *RORA* (**H**) expression levels of tumor and adjacent normal tissues in NSCLC patients. *P < 0.05, ****P < 0.0001

To further probe and validate the prognostic value of these five clock genes, we used univariate and multivariate cox regression analyses and established a nomogram model to predict the OS of NSCLC patients (Fig. 3A-D). Univariate Cox regression analysis revealed that among the five clock genes, *TIMELESS* (*P* = 0.034, HR = 1.19 [1.09, 1.32]) and *RORA* (*P* = 0.042, HR = 0.88 [0.78, 0.99]) were correlated greatly with the OS of NSCLC (Fig. 3A). Moreover, multivariate Cox regression analysis indicated that *TIMELESS* (*P* = 0.004, HR = 1.21 [1.06, 1.38]) and *RORA* (*P* = 0.047, HR = 0.868 [0.755, 0.998]) were independent prognostic factors for OS in NSCLC (Fig. 3B). Based on that, an OS nomogram model was established to estimate the overall survival of NSCLC patients with *TIMELESS* and *RORA* expression levels, and age (Fig. 3C). The points of *RORA*, *TIMELESS*, and age were added to obtain the total points, by which the OS rate in 1, 2, 3, and 5 years was predicted. The calibration curve (Fig. 3D) for the OS nomogram indicated a conceivable prediction effect of the model. These results indicated that *TIMELESS* and *RORA* had a high prognostic value in NSCLC.

We further explored the expression profiles of *TIMELESS* and *RORA* in different pathological TNM (pTNM) stages in NSCLC using data from TCGA database (Fig. 3E-F). TIMELESS expression in stage II NSCLC was much higher than that in stage I (*P* < 0.0001), while compared with stage I, RORA expression in stage III was significantly lower (*P* < 0.0001). Thirteen pairs of clinical NSCLC samples from the Third Affiliated Hospital of Kunming Medical University were used to confirm the expression pattern of TIMELESS and RORA using RT-qPCR methods. TIMELESS was significantly upregulated (Fig. 3G, P < 0.05) while RORA was significantly downregulated (Fig. 3H, P < 0.0001) in tumor tissue compared to the adjacent normal tissue.

### Genes and function related to *TIMELESS* and *RORA* in NSCLC TCGA samples

**Fig. 4:**
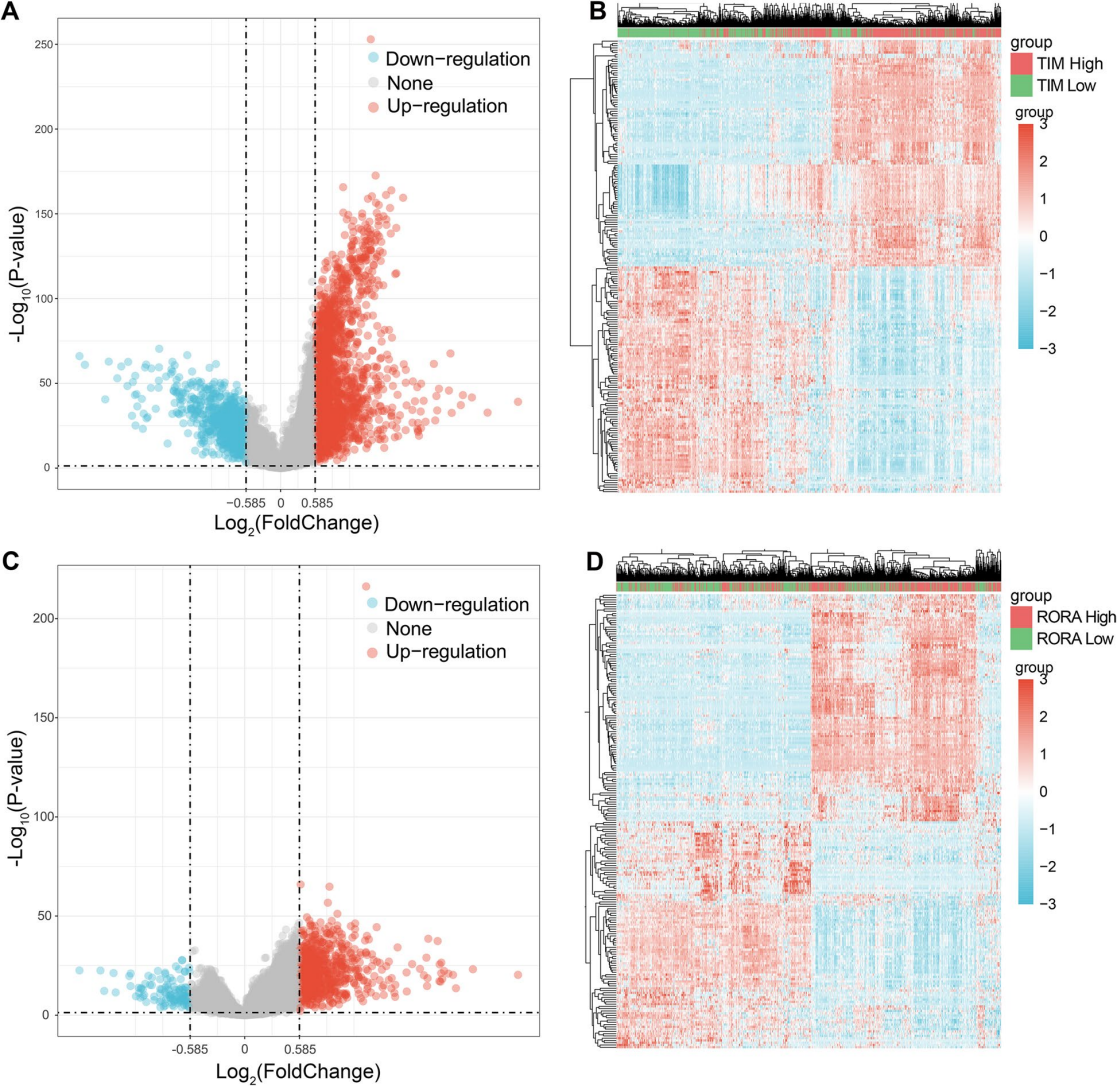
Identification of genes corelated to *TIMELESS* and *RORA* in NSCLC samples. Volcano plots were constructed using fold change and adjusted *P*-value to probe the genes significantly correlated to *TIMELESS* (**A**) and *RORA* (**C**) expression level. The red point in the plot represents the over-expressed mRNAs and the blue point indicates the down-expressed mRNAs with statistical significance. The hierarchical clustering analysis of mRNAs, which were significantly differentially expressed between high and low expression of *TIMELESS* (**B**) as well as *RORA* (**D**). Fold change > 1.5 and *P* < 0.05 were considered statistically significant

To investigate the biological function of *TIMELESS* and *RORA*, we used Limma R package to identify related genes of *TIMELESS* and *RORA*. The median of *TIMELESS* or *RORA* expression level was the basis by which the TCGA NSCLC samples were divided into two groups with a threshold of fold change > 1.5 and *P* < 0.05. Collectively, 1955 genes were significantly upregulated in the high *TIMELESS* expression group, indicating a significantly positive correlation with *TIMELESS*, whereas 799 genes were significantly negatively correlated with *TIMELESS* (Fig. 4A-B). Regarding *RORA*, 709 genes had significantly positive correlation, and 117 genes had a significantly negative correlation (Fig. 4C-D). The correlated genes are listed in Table S1.

**Fig. 5:**
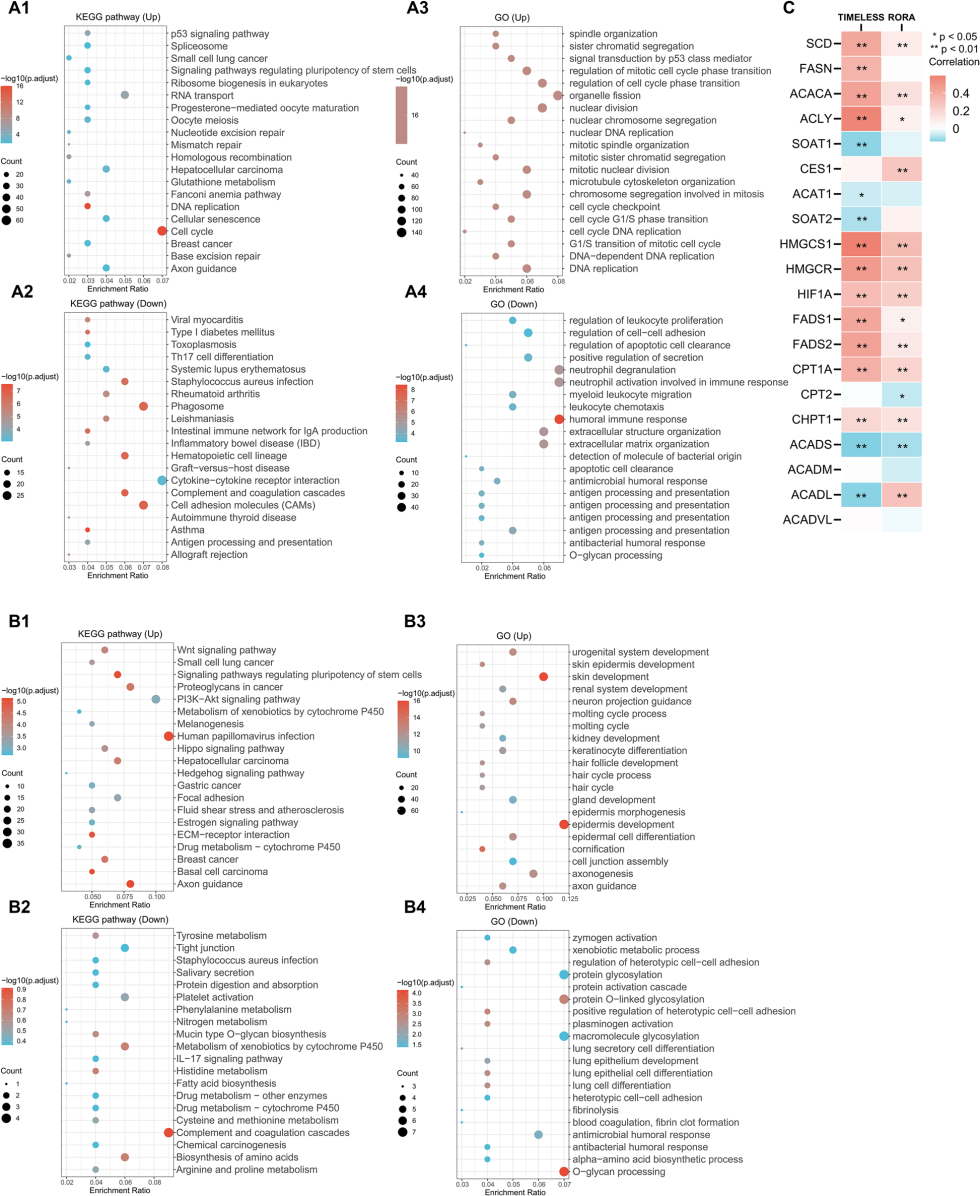
Functional enrichment analysis of genes significantly correlated with *TIMELESS* and *RORA* in NSCLC samples. KEGG and GO pathway enrichment analysis of *TIMELESS*-related (A1-A4) and *RORA*-related up and down genes (B1-B4). Only top 20 clustered were shown in each image. (C) Heatmap of the correlation of *TIMELESS* and *RORA* expression in NSCLC TCGA samples with lipid metabolism. Red means positively correlated and blue means negatively correlated to *TIMELESS* and *RORA* expression. *P < 0.05, **P < 0.01

GO and KEGG signaling pathway enrichment analyses were done for the upregulated or downregulated genes of *TIMELESS* (Fig. 5A1–A4) or *RORA* (Fig. 5B1–B4), respectively. Genes positively correlated with *TIMELESS* were mainly enriched in the cell cycle, and DNA replication. There are “mismatch repair” and “nucleotide excision repair” clusters in KEGG pathways and all clusters in GO, indicating the strong impact of *TIMELESS* on the cell cycle (Fig. 5A1–A2). Interestingly, the significantly downregulated genes were mainly enriched in the clusters strongly related to the immune system; for example, antigen processing and presentation, complement and coagulation cascades, and cytokine-cytokine receptor interaction were directly related to immunological reactions, while other KEGG clusters were related to hypersensitivity or autoimmune diseases (asthma, systemic lupus erythematosus, and inflammatory bowel disease so on; Fig. 5A3 and A4). These results indicate that *TIMELESS* might exert cancer-promoting effects by influencing multiple immunologic processes.

Regarding *RORA*, the upregulated clusters in KEGG pathways were complicated, including many cancers and signaling pathways (e.g., Wnt signaling pathway, PI3K-Akt signaling pathway, Hedgehog signaling pathway; Fig. 5B1), while the upregulated GO clusters were enriched in embryonic development. The downregulated KEGG pathway was unenriched (*P* > 0.05), and the downregulated GO clusters were mainly enriched in glycosylation and protein activation (e.g., macromolecule glycosylation, protein glycosylation, and protein activation cascade). Interestingly, some clusters were related to lung cell development and differentiation, which might indicate the tissue specificity of the effect of *RORA*.

The correlation of *TIMELESS* and *RORA* expression with lipid metabolism is shown in Fig. 5C. Many important molecules in lipid metabolism had a significant correlation with *TIMELESS* and *RORA* expression level (*P* < 0.05). For example, *SCD*, *FASN*, *ACACA*, *ACLY*, *HMGCS1*, *HMGCR*, *HIF1A*, *FADS1*, *FADS2*, *CPT1A*, and *CHPT1* were significantly positively correlated with *TIMELESS* expression, and *SOAT1*, *ACAT1*, *SOAT2*, *ACADS*, *ACADL* had a significantly negative correlation (*P* < 0.05).

### *TIMELESS* and *RORA* expression levels are associated with immune infiltration level in NSCLC

**Fig. 6:**
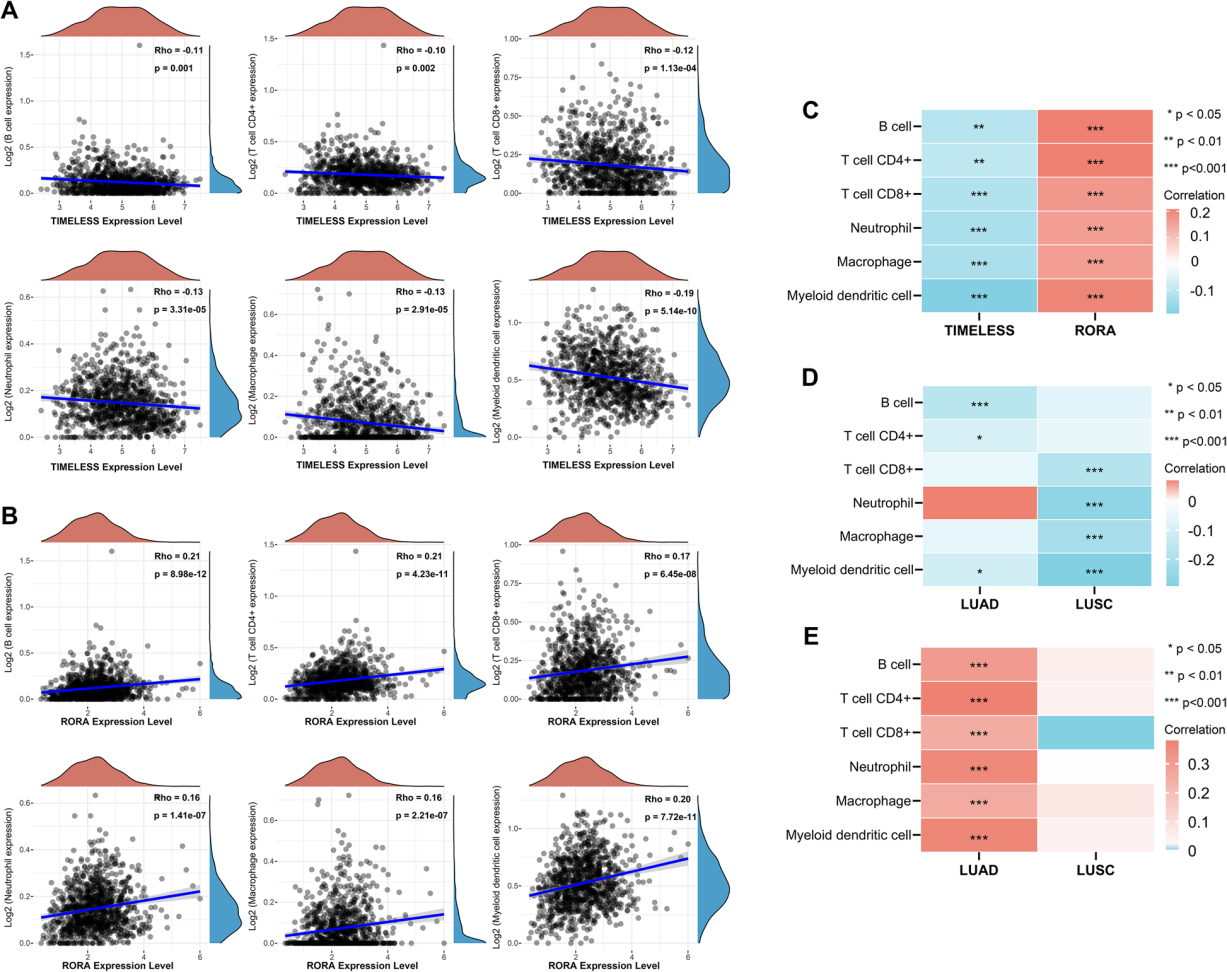
Correlation of *TIMELESS* and *RORA* expression level with immune infiltration in NSCLC. Spearman correlation analysis of *TIMELESS* (**A**) and *RORA* (**B**) expression with immune infiltration levels of CD8+ T cells, CD4+ T cells, Neutrophils, mDCs, Macrophages, and B cells in NSCLC was performed using the TIMER algorithm. The horizontal axis in the figure represents the expression and distribution of the gene, and the ordinate is the immune infiltration level. **C** Heatmap of immune infiltration levels of CD8+ T cells, CD4+ T cells, Neutrophils, mDCs, Macrophages, and B cells with *TIMELESS* and *RORA* expression in NSCLC. The correlation of *TIMELESS* (**D**) and *RORA* (**E**) expression with immune infiltration levels by “immunedeconv” package of R software. *P < 0.05, **P < 0.01, ***P < 0.001

As an independent predictor of the status of sentinel lymph node, tumor infiltrating lymphocytes have been gathering more and more attention; therefore, the probable correlation between *TIMELESS* and *RORA* expression and immune infiltration levels in NSCLC was assessed. We found that higher expression of *TIMELESS* led to the significant downregulation of B cells, CD4+ T cells, CD8+ T cells, neutrophils, macrophages, and mDC infiltration levels (*P* < 0.05; Fig. 6A, C). Whereas, *RORA* expression was positively correlated with all six types of immune cells in NSCLC (*P* < 0.05; Fig. 6B-C).

The results were further validated using the immunedeconv package of R software. Consistently, *TIMELESS* expression was also significantly negatively correlated with the infiltration of CD4+ T cells, mDCs, and B cells in LUAD, and CD8+ T cells, neutrophils, macrophages, and mDCs in LUSC (*P* < 0.05; Fig. 6D). Moreover, in LUAD, *RORA* expression was also significantly positively correlated with all six types of immune cells (*P* < 0.05; Fig. 6E), which was in good agreement with the results using the TIMER algorithm.

**Fig. 7:**
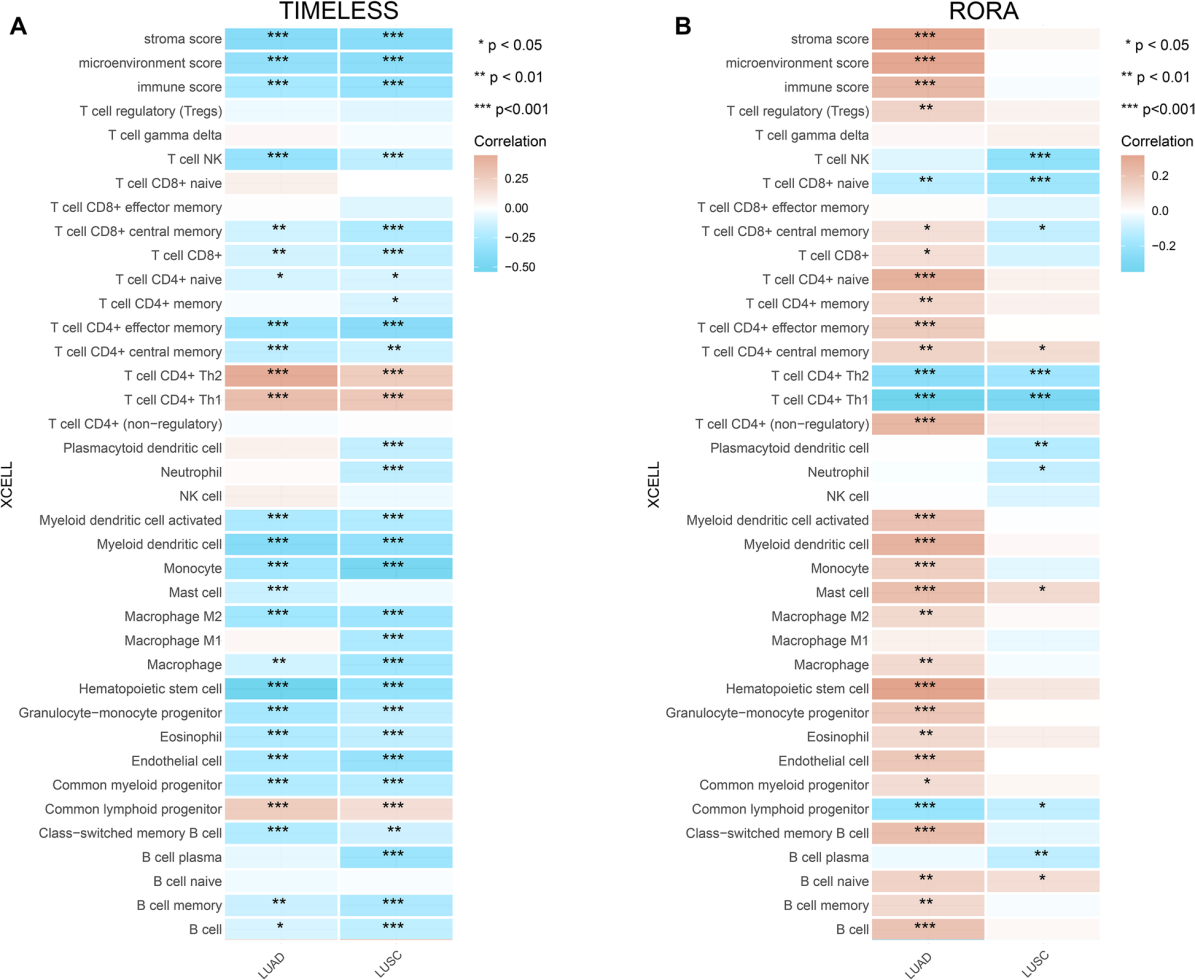
Correlation of *TIMELESS* and *RORA* expression level with immune infiltration in NSCLC using algorithm of XCELL database. Correlation of *TIMELESS* (**A**) and *RORA* (**B**) expression with immune infiltration levels using the algorithm of XCELL database. *P < 0.05, **P < 0.01, ***P < 0.001

To further assess the correlation, we examined the detailed infiltration level using the algorithm of XCELL database. The results showed that *TIMELESS* expression level was negatively correlated with most immune cell infiltration in both LUAD and LUSC (*P* < 0.05; Fig. 7A). By contrast, the *RORA* expression level was positively correlated with most immune cell infiltration in LUAD (*P* < 0.05; Fig. 7B), with no obvious correlation in LUSC.

### *TIMELESS* and *RORA* are associated with TMB and immune checkpoint-related genes

**Fig. 8:**
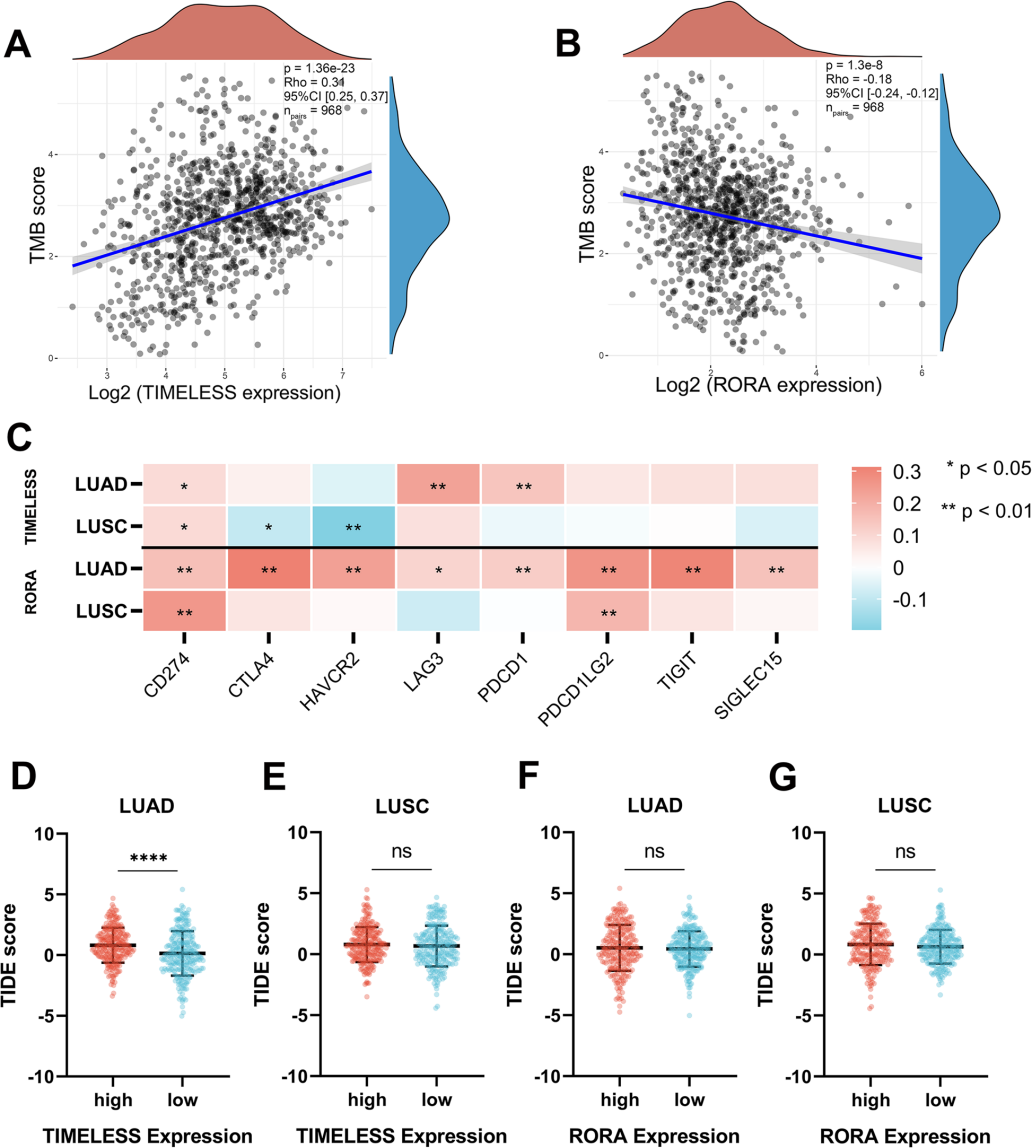
Correlation of *TIMELESS* and *RORA* expression level with TMB and immune checkpoint in NSCLC samples. Correlation analysis of *TIMELESS* (**A**) and *RORA* (**B**) expression with TMB. The horizontal axis in the figure represents the expression distribution of the gene, and the ordinate is the expression distribution of the TMB score. The density curve (blue) on the right represents the distribution trend of the TMB score, and the upper density curve (brown) represents the distribution trend of the gene. **C** Heatmap of the correlation of *TIMELESS* and *RORA* with immune checkpoint-related genes in LUAD and LUSC samples. Red means positive correlation and blue means negative correlation. **D**-**G** The TIDE score of NSCLC patients with different *TIMELESS* or *RORA* expression. *P < 0.05, **P < 0.01, ****P < 0.0001

To further assess the influence of *TIMELESS* and *RORA* on the effect of immune checkpoint inhibitors, the TMB was investigated. There was a significant positive correlation between *TIMELESS* expression and the TMB score in NSCLC (*P* < 0.0001, ρ = 0.31 [0.25, 0.37]; Fig. 8A). Also, the TMB score was negatively correlated with *RORA* expression in NSCLC (*P* < 0.0001, ρ = − 0.18 [− 0.24, − 0.12]; Fig. 8B).

We also examined the association of *TIMELESS* and *RORA* with immune checkpoint-related genes (Fig. 8C). *TIMELESS* expression was positively correlated with *CD274*, *LAG3*, and *PDCD1* in LUAD as well as *CD274* in LUSC significantly (*P* < 0.05), and was negatively related to *CTLA4* and *HAVCR2* in LUSC (*P* < 0.05). *RORA* was significantly positively correlated with those immune checkpoint-related genes (*CD274*, *CTLA4*, *HAVCR2*, *LAG3*, *PDCD1*, *PDCD1LG2*, *SIGLEC15*, and *TIGIT*) in LUAD as well as with *CD274* and *PDCD1LG2* in LUSC (*P* < 0.05).

ICB therapy of cancer can bring various benefits. The response to this treatment of NSCLC patients with different *TIMELESS* or *RORA* expression were predicted by TIDE algorithm (Fig. 8D-G). In LUAD, the high expression of *TIMELESS* was correlated with a significantly higher TIDE score compared to patients with lower *TIMELESS* expression (Fig. 8D).

### The mutation of *TIMELESS* and *RORA* and its influence on immune cell infiltration in NSCLC

**Fig. 9:**
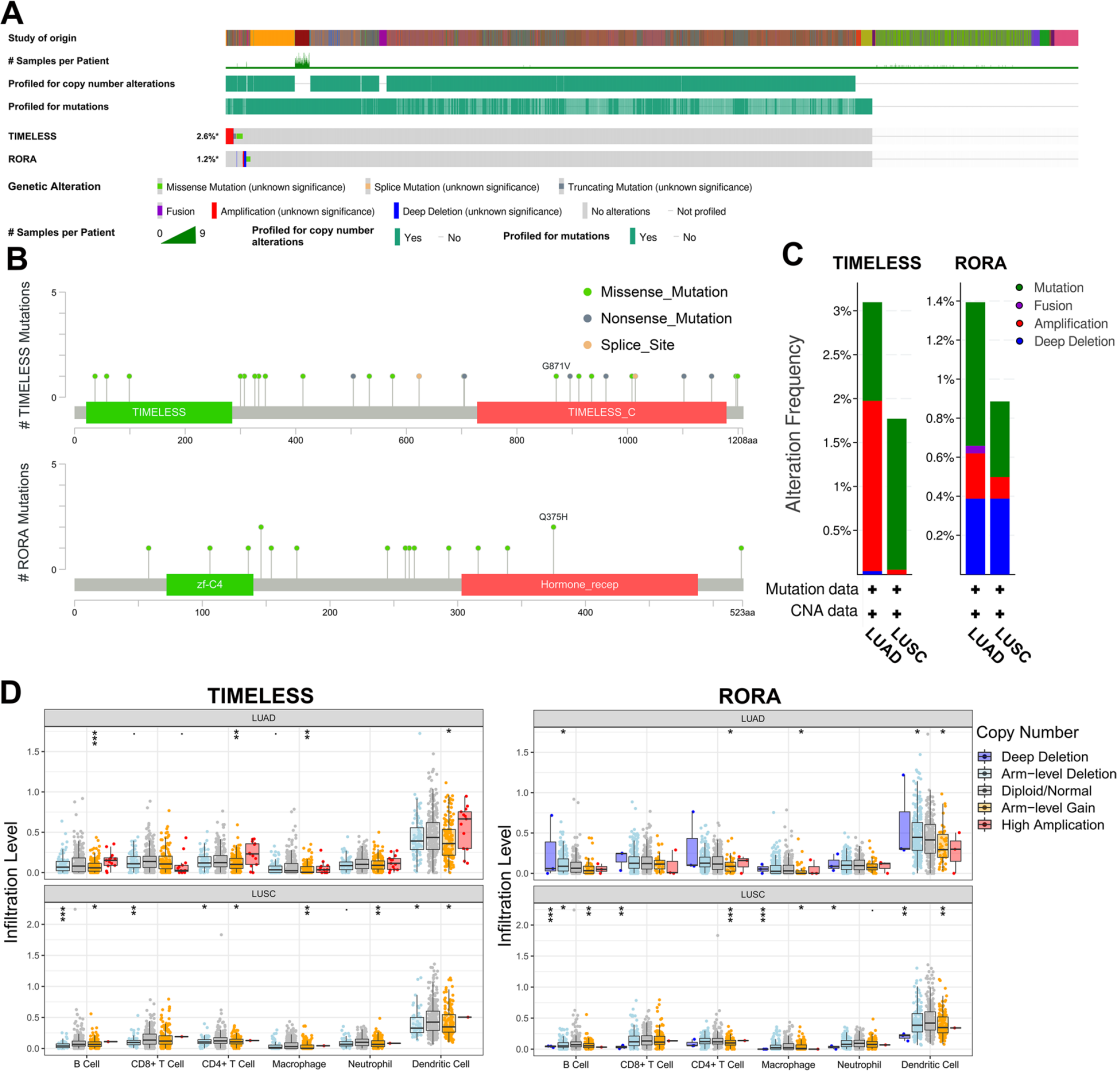
The mutation of *TIMELESS* and *RORA* and its influence on immune infiltration level. **A** The mutation frequency of *TIMELESS* and *RORA* in NSCLC samples analyzed by cBioportal database. **B** the lollipop plots showing the mutation map of *TIMELESS* and *RORA*. Green, gray and orange dots mean missense mutation, nonsense mutation, and splice site, respectively. **C** The histogram of *TIMELESS* and *RORA* alteration frequency in LUAD and LUSC. **D** The immune infiltration level correlated to different types of *TIMELESS* and *RORA* alteration (deep deletion, arm-level deletion, normal, arm-level gain and high amplification). *P < 0.05, **P < 0.01, ***P < 0.001

We analyzed the mutation situation of *TIMELESS* and *RORA* in NSCLC. The whole mutation rates of *TIMELESS* and *RORA* were 2.8 and 1.2% in NSCLC samples with cBioportal database, respectively (Fig. 9A), and the gene mutation type detected in *TIMELESS* included missense and nonsense mutations, while in *RORA*, the detected mutation consisted of only missense mutations (Fig. 9A-B). The detailed alteration status of the two clock genes is shown in Fig. 9C. Regarding *TIMELESS*, the alteration rate was higher in LUAD (3.1% of 2583 cases) than in LUSC (1.77% of 1087 cases). In LUAD, the two major alteration types were amplification (1.94%) and mutation (1.12%), while in LUSC, the amplification rate was quite rare (0.06%) and mutation was the major alteration type (1.72%). Regarding *RORA*, the alteration rate was lower (1.39% in LUAD and 0.89% in LUSC). Also, the dominant alteration type was mutation (0.74%) in LUAD, while in LUSC, the deep deletion rate (0.39%) was the same as the mutation rate.

Then the correlation between copy-number-change and the immune cell infiltration levels was analyzed using the TIMER database (Fig. 9D). The arm-level gain of *TIMELESS* in LUAD and LUSC was in a significant correlation with most immune cell infiltration (*P* < 0.05). Regarding *RORA*, a significant negative correlation of deep deletion and arm level-gain with immune infiltration level was observed (*P* < 0.05).

## Discussion

This study identified two important clock genes, *TIMELESS* and *RORA*, which were shown to have some relationship with NSCLC development and progression using bioinformatics analysis. *TIMELESS* mRNA and protein expression were upregulated, while the *RORA* expression were downregulated in NSCLC tissues. NSCLC patients with higher expression of *TIMELESS* or lower expression of *RORA* had a significantly poorer prognosis. The importance of the two genes in NSCLC was probed furtherly. Moreover, *TIMELESS* and *RORA* expression levels were two independent prognostic factors for OS in NSCLC. Also, we observed that TIMELESS expression in stage II NSCLC was higher than those in other stages and RORA expression in stage III was significantly lower compared to those at stage II or I.

Our findings with many independent datasets including thousands of samples are consistent with previous studies. Yoshida et al. found high *TIMELESS* expression levels in 22 lung cancer cell lines and 88 lung cancer samples, suggesting the potential of *TIMELESS* in diagnosis and prognosis of lung cancer [24]. The upregulation of *TIMELESS* has also been found in cervical carcinoma [25], colorectal cancer [26], and breast cancer [27], and the downregulation of *RORA* has been found in many cancers including hepatocellular carcinoma [28], gastric cancer [29], melanoma [30], and breast cancer [31]. In present study, we found that *TIMELESS* and *RORA* were two prognostic biomarkers in NSCLC. *TIMELESS* had tumor-promoting effects, and *RORA* had tumor-suppressing effects.

Accumulating evidence has shown that as a systemic disease, cancer involves the disorder of pathways and homeostasis in many aspects [32, 33]. Therefore, it is necessary to probe the role *TIMELESS* and *RORA* play in NSCLC systematically [34]. To further explore their functions, we identified genes that were significantly correlated with *TIMELESS* and *RORA* and found that genes positively correlated with *TIMELESS* expression were dramatically enriched in pathways related to the cell cycle and DNA replication. *TIMELESS* might interact with the eukaryotic elongation factor 1A2, which plays a role in cell migration and ribosomal protein biosynthesis [35]. Depletion of *TIMELESS* results in dysfunction of mitotic progression, impaired sister chromatid cohesion, and chromosome fragmentation [36]. Also, overexpression of claspin and *TIMELESS* can stabilize the replisome to reduce the chronic stress of replication on forks [37]. These data indicate the tumor-promoting effect of *TIMELESS* by promoting the cell cycle and DNA replication.

Interestingly, the significantly negatively correlated genes with *TIMELESS* were mainly enriched in the clusters strongly related to the immune system, antigen processing and presentation, complement and coagulation cascades, and rheumatoid arthritis. This suggests *TIMELESS* may take part in tumor immunological processes and exert inhibitory effects on tumor immunity. Therefore, we also discussed the relationship between *TIMELESS* and tumor immune function.

As an emerging independent predictor of sentinel lymph node status and tumor metastasis, tumor infiltrating lymphocytes has attracted considerable attention [38, 39]. So, we examined the potential correlation of *TIMELESS* and *RORA* expression with the immune infiltration levels in NSCLC by multiple algorithms (TIMER, “immuneeconv” R package, and XCELL). The negative correlation between *TIMELESS* expression and infiltration levels of most immune cells was observed according to TIMER and R package analysis, while the XCELL algorithm indicated a significant positive correlation of TIMELESS expression level and CD4+ Th1, Th2 cells. And *RORA* expression was positively correlated with most immune cell infiltration especially in LUAD. The immune infiltration levels of different algorithms were broadly in line with each other, especially for *TIMELESS* in LUSC and *RORA* in LUAD. These results were broadly consistent with functional enrichment analyses, indicating the importance of *TIMELESS* and *RORA* in the recruitment and regulation of immune infiltrating cells in NSCLC.

As an innovative therapy, considerable attention has been paid to immunotherapy in cancer treatment. Immune checkpoint inhibitors (mainly *PD-1/PD-L1* targets) have shown considerable advantages for several tumor types, especially NSCLC [40]. Therefore, it is of great significance to evaluate the effects of *TIMELESS* and *RORA* on ICBs. A high TMB is likely to increase the capacity of a tumor to generate neoantigens. The increased productions of neoantigens would be expected to render a tumor more immunogenic, thus increasing its likelihood of responding to immunotherapy. TMB may be characterized concerning illustration the aggregate number for physical mutations for every megabase or those nonsynonymous transformations in tumor tissues, including supplanting and insertion erasure changes, and has become a preferable biomarker to foresee the light of anti-*PD-1/PD-L1* therapy [41, 42]. Higher TMB is associated with higher immunogenicity and response to ICBs in lung cancer [43], and some meta-analyses also indicated a strong positive correlation [42, 44]. Interestingly, our results illustrated that there were significant correlations of TMB score with *TIMELESS* (positive) and *RORA* (negative) expression, indicating that *TIMELESS* and *RORA* expression level could estimate the *PD-1/PD-L1* blockers therapy response of patients as biological markers in some degree. We also found that *TIMELESS* was significantly positively related to *CD274*, *LAG3*, and *PDCD1* in LUAD as well as *CD274* in LUSC, and negatively related to *CTLA4* and *HAVCR2* in LUSC. *RORA* was significantly positively correlated with all immune checkpoint-related genes in LUAD and *CD274* and *PDCD1LG2* in LUSC. The tumor immune dysfunction and exclusion (TIDE) algorithm was mainly utilized to predict ICB response in NSCLC and melanoma, in which multiple gene expression markers were used to estimate the dysfunction and exclusion of tumor infiltration cytotoxic T lymphocytes (CTLs) [21]. In this study, LUAD patients with high *TIMELESS* expression had a significantly higher TMB score than those with low *TIMELESS* expression, indicating a better response to ICB therapy and more benefits. Collectively, these results indicated the probable potential of *TIMELESS* and *RORA* as predictors on the response to ICB therapy. However, as the therapy details of the data we used in those databases was not so clear, there are no direct clinical data to confirm the prediction effect of *TIMELESS* and *RORA*. The specific prediction effects of them, as well as the correlation of those factors require much further clinical exploration.

Tumor lipid metabolism has attracted extensive attention recently. Aberrant lipid metabolism is found in multiple human cancers [45], and lipid synthesis is essential for neoplastic growth in many cancer types [46]. More importantly, some molecules in lipogenesis have been identified to have tumor-promoting roles such as tumor invasion, metastasis, and other malignant biologic function. For example, fatty acid synthase (*FASN*) was upregulated in multiple cancer types [47] and is correlated with the amplification of *HER2* in breast cancer [48]; also, ATP-Citrate Lyase (*ACLY*) was upregulated in gastric adenocarcinoma [49], and downregulation of *ACLY* could decrease cell proliferation and invasion in multiple cancer cells [50]. We specifically examined the correlation of *TIMELESS* and *RORA* with lipid metabolism. The results indicated that molecules in lipid metabolism including *SCD*, *FASN*, *ACACA*, *ACLY*, *HMGCS1*, *HMGCR*, *HIF1A*, *FADS1*, *FADS2*, *CPT1A*, and *CHPT1* were strongly positively correlated with *TIMELESS*. *TIMELESS* upregulated *ACER2* expression and mitochondrial respiration thus increased S1P synthesis in ER-positive breast cancer and is closely correlated with poor prognosis [51].

In addition, the immune cells in tumor microenvironment also have abnormal lipid metabolism and cause abnormal tumor immunity. Nevertheless, there is a correlation between abnormal lipid metabolism and tumor immunity. *TIMELESS* maybe the key gene related to tumor and immune and lipid metabolism. Or it is possible to find an effective recognition of the metabolic pathways shared between tumor and immune cells through *TIMELESS*, therefore targeting tumor metabolism and reprogramming immune cell metabolism.

## Conclusion

Collectively, we identified two genes, *TIMELESS* and *RORA*, as the key clock genes in NSCLC. Their importance in the pathogenesis of NSCLC was proved from the perspective of prognosis and gene expression. Moreover, using bioinformatics analysis, we probed the function of *TIMELESS* and *RORA* in multiple aspects, especially in tumor immune regulation and *TIMELESS* in lipid metabolism. These results indicate the strong potential of *TIMELESS* and *RORA* to be the biomarkers for NSCLC progression and prognosis.

## Supplementary Information

**Additional file 1: Table S1**. Genes significantly related to TIMELESS and RORA.

## Data Availability

The data of the current study is available from the following open public databases: TCGA (https://cancergenome.nih.gov), Oncomine (https://www.oncomine.org/), and GTEx (https://gtexportal.org/home/datasets) as is described above. Other data will be available from the corresponding author upon reasonable request.

## Abbreviations

NSCLC: Non-small cell lung cancer
*PER1*: Period circadian regulator 1
*PER2*: Period circadian regulator 2
*CRY1*: Cryptochrome circadian regulator 1
*CRY2*: Cryptochrome circadian regulator 2
*TIMELESS*: Timeless circadian regulator
*RORA*: Retinoic acid-related orphan receptor A
*NR1D1*: Nuclear receptor subfamily 1, group D member 1
*NR1D2*: Nuclear receptor subfamily 1, group D member 2
TNM: Tumor-node-metastasis
LUAD: Lung adenocarcinoma
LUSC: Lung squamous cell carcinoma
TCGA: The Cancer Genome Atlas
GTEx: Genotype-Tissue Expression
GO: Gene Ontology
KEGG: Kyoto Encyclopedia of Genes and Genomes
TMB: Tumor mutation burden
TIDE: Tumor immune dysfunction and exclusion
ICB: Immune checkpoint blockade
CTLs: Cytotoxic T lymphocytes
TIMER: Tumor immune estimation resource
mDCs: Myeloid dendritic cells
OS: Overall survival
PFS: Progression-free survival
PPS: Post-progression survival
*FASN*: Fatty acid synthase
*ACLY*: ATP-Citrate Lyase
