# Supplementary material for: Identification of TIMELESS and RORA as key clock molecules of non-small cell lung cancer and the comprehensive analysis
Source: BMC Cancer. 2022 Jan 25;22:107. doi: 10.1186/s12885-022-09203-1 (PMC8788117; doi:10.1186/s12885-022-09203-1)
Supplement: Supplementary file 1 — Additional file 1: Table S1. Genes significantly related to TIMELESS and RORA. [file 12885_2022_9203_MOESM1_ESM.pdf]

Table S1 Genes significantly related to TIMELESS and RORA

TIMELESS		RORA	
Up	Down	Up	Down
ESPL1	HOPX	TTBK2	GALE
CDK2	ST3GAL5	TCF4	SLC39A4
KIF18B	SFTA2	TSHZ2	KRT18
NCAPH	C16orf89	SLCO3A1	TLCD1
FANCI	C1orf116	PHC3	C4orf48
FOXM1	SLC22A31	HECA	S100P
CDC25A	NPC2	SLC9A9	KRT7
TICRR	CAPN8	SETBP1	GPR39
RAD54L	NAPSA	SEMA6D	SDSL
TROAP	HNF1B	BACH2	STARD10
CENPO	FOLR1	CACNA2D3	PYCR1
KIF23	MUC1	FOXO1	C11orf86
CHAF1A	SFTA3	MYO5A	SPDEF
UNG	CFAP221	PIK3CA	SMIM22
CDCA5	GGTLC1	DIPK2A	REEP6
RACGAP1	ACSL5	LEF1	SMPDL3B
PRC1	PNMA2	FGFR2	FAM83A
RFC5	RORC	MAF	B3GNT3
MCM2	STEAP4	SDK1	MROH6
BLM	MUC21	PTPRD	KPNA7
BUB1B	LMO3	EML1	TJP3
ORC1	FMO5	FOXN3	CRACR2B
NCAPD2	SLC34A2	PGAP1	TMEM92
E2F7	TMEM125	PLEKHG1	HGD
KIF4A	SLC44A4	VPS8	MISP
NEMP1	BCL2L15	MCC	GOLT1A
TPX2	FCER1A	SLC12A6	C19orf33
POLQ	SCN7A	TENM4	CLDN3
MCM7	TMEM163	SOX5	WFDC3
SPAG5	NKX2-1	TUB	ALDH3B1
EME1	GLOD5	PELI2	PHLDA2
AUNIP	RNASE1	ATP11B	QPRT
FEN1	GPR39	GLI2	ICA1
BUB1	CISH	MYCBP2	VSTM2L
KIFC1	ACOX2	BICD2	PLEK2
CDC45	ARL6IP5	NXPE3	PPP1R14D
EXO1	ADGRF5	EPB41L2	AGR2
CDC6	ALDH3B1	KLF12	MYEOV
CDCA3	SLC26A9	SDK2	NPW
PSMC3IP	RBPMS	C20orf194	CYP24A1
RFC4	HNMT	SIPA1L2	ISG15
C17orf53	CEACAM6	WNT5A	RORC

HASPIN	CD1C	ITPKB	MB
GTSE1	FBP1	TBX3	PON3
SKA1	STK32A	TLE4	TRIM31
KIF11	MLPH	ACAP2	SYT12
KIF15	SPDEF	EDDM13	CNTD2
BRCA1	AGR3	SESN3	SFTA2
MCM4	CRIP1	ARHGAP24	SNCG
MCM10	SLC22A3	ADAM23	LRG1
TTK	ARHGEF38	ARHGAP28	MSLN
NUSAP1	SMIM6	HDGFL3	TFPI2
RAD51AP1	GNA14	ABCC5	MUC1
MCM8	CD55	SPARCL1	KCNN4
DTL	CTSH	UBXN7	MGST1
KIF14	TMEM92	P2RY1	CDA
INCENP	DPP4	NTRK2	B3GNT6
PLK4	C1QTNF7	KLHL24	TMEM125
ARHGAP11A	PRR15L	CACNA2D1	MUC21
PLK1	NINJ2	CLDN20	FBXO2
NDC80	PDZK1IP1	RNF217	CST6
KNL1	SELENBP1	NEO1	FA2H
OIP5	SMIM22	RCAN1	AKR7A3
CDT1	SGMS2	ABCA5	BPIFA2
DDIAS	RAB27A	SMARCA2	HNF1B
CENPA	NDNF	RNF168	FGB
TOPBP1	TMPRSS2	NHSL1	BCL2L15
XRCC2	ALOX15B	SLIT2	PAEP
AURKB	DLC1	CACHD1	TESC
CKAP2L	NME5	TSPAN18	TMEM45B
CDCA8	UBXN10	PCYT1B	TNNC2
CCNB2	HLA-DMA	BCL11B	CCNJL
ZWILCH	CFI	FAM43A	HPN
MMS22L	CTSE	DLG1	DUSP4
NCAPG	PIGR	METTL24	RGL3
CENPF	ATOH8	PRKX	C15orf48
RAD51	MGP	MID2	FGA
SGO1	MGLL	ADD2	EPHX3
ORC6	HPGDS	CTTNBP2	PLEKHA6
UHRF1	INMT	KLHL42	CLDN6
SPC25	ARHGDIB	PLCB4	C4BPB
CLSPN	TMC5	ZEB1	KCNK5
HJURP	TNFRSF10C	ROBO2	PRR15L
FAM72B	FAM20A	TMTC1	FGL1
LMNB2	SCTR	GPC3	METTL7B
ZNF367	PLAC9	PCSK5	TMC5

CHEK1	GNG7	TTC14	EREG
SKA3	RPS6KA2	DIP2B	GCNT3
BRIP1	MVP	FRAS1	FOLR1
NCAPG2	CDKL2	COLGALT2	GPRC5A
CIP2A	VSIG2	NAV1	FOXJ1
HELLS	EMB	KIAA1671	MLPH
SENP1	SMPDL3B	ABCA13	PDZK1IP1
ASPM	KCNK5	PTCH1	SLC22A31
TOP2A	C4BPA	MERTK	ACSL5
ZWINT	KCNQ3	FZD7	KRT81
LMNB1	SELENOW	SVEP1	SLC44A4
DKC1	MYBPHL	GOLIM4	NKX2-1
FAM72A	SUSD2	NAP1L2	BHMT2
POLA2	PON3	DZIP1L	AOC1
SKP2	SFTPB	CHST7	FGG
MTFR2	C9orf152	DST	MUC13
CENPI	SYNE1	USP34	DNAJC12
KIF2C	CDH15	TET3	TFF1
CDCA2	TREM1	PPM1L	CPS1
FANCD2	PTPRE	AMOTL1	ARSE
SMC2	FCHO2	STON1	AQP3
SMARCD1	SDR16C5	YEATS2	MUC5B
PARPBP	IGIP	PLA2R1	CAPN8
TMPO	SERPINA1	MDGA1	LCN2
CENPE	PLA2G10	SYNM	SPINK1
FANCB	CYB5A	GFOD1	CEACAM6
MSH6	SLC4A4	STOX2	NAPSA
POLE	SNED1	COL4A5	SFTA3
TRAIP	SLC16A4	PCYT1A	EEF1A2
ANAPC7	RFTN1	SLC7A8	BPIFA1
EZH2	RHOBTB2	RUNX3	AZGP1
ECT2	CXCL17	VLDLR	
WDR62	MEF2C	PRIMA1	
TYMS	AK1	ZNF462	
DSCC1	COL4A3	NDRG4	
CCNA2	C10orf95	LRRC4	
ERCC6L	ROS1	PCDH19	
KPNA2	CCDC68	PAK5	
SUV39H1	BOK	JAKMIP2	
MCM3	ADGRD1	ABCA1	
FAM83D	AQP7	ZNF770	
MCM6	WFDC2	BOC	
WDHD1	GPRC5A	SORL1	
MYBL2	CXXC5	PIK3R1	

PRIM1	SCNN1B	DMRT2	
PRR11	MMP28	DENND2C	
KNTC1	ABCC6	MICAL3	
GINS1	CD1E	ARHGEF26	
GINS4	NFE2	NRCAM	
CSE1L	RILP	VANGL2	
PIMREG	TMEM150A	SOGA1	
CHAF1B	IL6ST	ARHGEF28	
SPC24	CLDN23	DACH1	
ATAD5	SPINK1	EHD3	
CENPH	CCNJL	COBLL1	
KNSTRN	AQP1	SCD5	
ASF1B	SLC9A3R2	BCHE	
MTBP	SLC49A3	JMJD7-	
SRSF9	TGFBR2	PLA2G4B	
PCNA	NOSTRIN	HEG1	
XPOT	PIFO	TSLP	
FBXO5	CLIC3	RNF152	
MKI67	AHCYL2	CRMP1	
LSG1	SFXN3	WNT2B	
GMPS	KLF2	PHLDB2	
MELK	SMIM14	PALLD	
E2F2	SLC39A8	ATP2B1	
SART3	CHI3L2	GJA1	
KIF18A	HABP2	MARK1	
DEPDC1	GAPT	MAP1B	
RFWD3	SHE	SOX2	
NUP205	LY86	JARID2	
AURKA	IFITM2	PRR5L	
NUF2	HPGD	GCSAM	
NEK2	LONRF3	GLI1	
ESCO2	SFTPD	DUOX1	
PSRC1	CHIA	GPNMB	
STIL	KLHDC7A	EPHB6	
CDC20	CCDC69	BLMH	
BIRC5	EMCN	GHR	
MGME1	PLA2G1B	DVL3	
KMT5A	CST5	MYH11	
CDC7	GOLT1A	TGFBR3	
KIF20A	TSPAN4	AKT3	
TRA2B	GJB1	NPL	
CCNF	SOD3	GSTM2	
TRIP13	DNALI1	NFE2L2	
MCM5	FOXA2	NMNAT3	

GINS3<br>IMMT<br>DBF4B<br>UBE2S<br>CDK1<br>SGO2<br>PKMYT1<br>NCAPD3<br>DLGAP5<br>TEDC2<br>VRK1<br>MAD2L1<br>RANBP1<br>MARS<br>PSMD11<br>ATAD2<br>GINS2<br>RRM2<br>DENR<br>RFC3<br>CENPM<br>RBL1<br>PTBP1<br>PBK<br>PA2G4<br>ZFP64<br>PRPF19<br>SSRP1<br>MIS18A<br>RNASEH2A<br>FANCG<br>TCF19<br>FANCA<br>DEPDC1B<br>LIG1<br>TBC1D31<br>TDG<br>MASTL<br>TACC3<br>RMI2<br>PSMD2<br>R3HDM1<br>REPS1<br>CHEK2	LRRN4<br>CELF2<br>ARRB1<br>LRG1<br>CPM<br>SEC14L6<br>CITED2<br>ITGA8<br>CST6<br>APOH<br>STS<br>MMP24<br>TNFSF12<br>ZMYND12<br>CRYM<br>CTSO<br>SLC46A2<br>CST3<br>FAM107B<br>CYSLTR1<br>AGRP<br>MBIP<br>CYP4B1<br>LIMCH1<br>ADH1B<br>TCF21<br>CX3CR1<br>PEBP4<br>SNTB1<br>SCEL<br>ARRDC3<br>VSTM2L<br>MSLN<br>GNG11<br>KRT7<br>HSPB7<br>HLA-DPB1<br>PAG1<br>CCDC170<br>MAP1LC3C<br>ARSE<br>AGR2<br>TRAM1<br>GLRX	PLD1<br>LSAMP<br>AASS<br>MAPKBP1<br>CBX6<br>MRAP2<br>MCOLN3<br>IGSF3<br>C21orf91<br>JAG1<br>PCDHGC3<br>TMEM117<br>MAPK10<br>PTPN14<br>EGR2<br>TMPRSS11F<br>MATN2<br>KCNJ2<br>NRN1<br>SLC2A12<br>FGF2<br>KIRREL1<br>FAM117B<br>SYNGR1<br>PTPRZ1<br>HAS3<br>EIF5A2<br>DKK3<br>EGFL6<br>RASSF9<br>RGMA<br>ADD3<br>FXYD6<br>OLFM1<br>SEMA6A<br>SLC35G1<br>TP63<br>STON2<br>TMTC3<br>GPR161<br>MAP1A<br>KLHL13<br>BCL11A<br>TMEM246	

TTF2	ASAH1	ABCC1	
RRM1	HLA-DRB1	RAET1L	
SASS6	DRAM1	PDZRN3	
BORA	SLC1A7	PYGO1	
DNAJC9	ORM2	INSYN1	
CKAP2	S100B	NFIX	
NOP56	FAM81B	AHNAK	
DNA2	SIAE	ITGB8	
EXOSC2	TMEM45B	GBP6	
SMC4	CERKL	SCARA3	
HNRNPM	ESYT3	ZBTB7C	
CEP55	AQP3	ADH7	
NUP37	HLA-DQB1	CD44	
C1orf112	RCAN2	SGK1	
CEP152	GLB1L3	PTPRS	
DBF4	PLA2G12B	WNT11	
EFTUD2	SLC46A3	UST	
YEATS2	RAB17	IGSF10	
CENPN	ATP8A1	LRP1	
ZBTB39	ORM1	CREB5	
SNRPD1	SORCS2	LIN7A	
GEN1	PGC	SMOC2	
HDAC2	ARHGAP40	LGALSL	
RNASEH1	JAML	ARHGEF25	
CEP78	LGALS3	AUTS2	
KHSRP	CD302	CHST2	
RCC2	EPHA10	CDH13	
GMNN	CD74	SMO	
FBXO45	KNDC1	KLF4	
DSN1	HLA-DRA	F2RL2	
TFCP2	GALNT10	CRYAB	
CCDC77	LST1	CPXM2	
CAD	HIGD1B	BMP7	
TIPIN	PLD4	DLX5	
CCNB1	NR3C2	FEZ1	
DVL3	RETN	THEMIS	
RPA1	SCGB3A1	DCUN1D1	
TMEM201	SYTL2	ADAMTS1	
OPA1	C3	CDHR1	
RAE1	ACOXL	TMCC2	
NUP107	B3GNT8	DIAPH2	
FAM72D	PAM	PAMR1	
SMPD4	TXNIP	DGKA	
TET3	FHL5	LAMA2	

NOP2<br>TUBA1B<br>RECQL4<br>ACTL6A<br>PSME4<br>PIF1<br>SNRPA<br>HAUS8<br>MND1<br>AAAS<br>UBE2T<br>HMGA1<br>PRIM2<br>PPM1G<br>BRI3BP<br>DNMT1<br>CENPW<br>ATP5F1B<br>NOL10<br>SNRPA1<br>ANLN<br>KDM2B<br>KIF22<br>WDR43<br>SHMT2<br>CEBPG<br>CBX1<br>ADIPOR2<br>DIAPH3<br>UCK2<br>FXR1<br>SPINDOC<br>TK1<br>NDC1<br>E2F1<br>RUVBL1<br>SAE1<br>SNRPF<br>POLA1<br>XPO1<br>RHNO1<br>ALYREF<br>H2AFZ<br>CTPS1	PARM1<br>DOK4<br>TNNC1<br>HSD17B11<br>CD151<br>ELFN2<br>PGM5<br>IL37<br>ATP11A<br>ITGB6<br>SCGB3A2<br>BEAN1<br>NRP1<br>SORBS2<br>HLA-DQB2<br>DDAH1<br>PDE4C<br>FAXDC2<br>ATP10A<br>B3GNT6<br>GPD1L<br>SHROOM4<br>SPINK13<br>TDRD10<br>ABCC3<br>MFSD4A<br>ADAMTS8<br>RGN<br>ZNF69<br>MACC1<br>KIF12<br>ANG<br>A2M<br>CFB<br>GMFG<br>EPDR1<br>TJP3<br>ENPP4<br>VEPH1<br>S100A4<br>CD47<br>ITGA9<br>CRIP2<br>RGCC	BTG2<br>NOTCH1<br>LTB4R2<br>FETUB<br>IGF1R<br>CEP19<br>BCL2<br>EMP1<br>SHROOM2<br>CSRP2<br>MN1<br>OXGR1<br>LIMK2<br>NPR2<br>ARRDC4<br>LRATD1<br>ABCG1<br>CNTNAP2<br>ITGA6<br>TENM2<br>DAPL1<br>XXYLT1<br>CACNA1B<br>VSNL1<br>TACC1<br>CALML3<br>PHF24<br>DMRT3<br>PTGFRN<br>TSPAN7<br>LGI3<br>HAP1<br>FAT2<br>SAMD12<br>COL4A6<br>HMCN1<br>SBSPON<br>HEY1<br>FREM2<br>TRPV4<br>NPTXR<br>PTN<br>UGT1A7<br>FRMD6	

BRCA2	HLA-DRB5	LARGE1	
MTA2	DNAJB13	SOSTDC1	
SCML2	DPYSL2	DUOXA1	
WBP11	LPCAT1	NELL2	
CDC25C	ICAM1	FBLN1	
USP5	TMEM273	CELSR2	
WDCP	CLIC5	CLCA2	
SSX2IP	SMIM1	RAI2	
C19orf48	HKDC1	PTGER4	
DNAJB11	EVA1A	RADX	
MTIF2	ESAM	COPZ2	
HAT1	B3GNT7	LAYN	
CENPU	NNMT	DPYSL4	
CKAP5	GPX3	PLXDC2	
DHX9	RHOU	ACP7	
DNM1L	MALL	EMILIN3	
CDCA4	TSC22D3	GABRE	
THOP1	HLA-DPA1	LTB4R	
MSH2	BTBD9	NRG1	
SRRT	KCNQ1	KCTD15	
SMC1A	SULT1A2	PGBD5	
UMPS	SLC44A3	MAFB	
SET	COL4A4	MYLK	
XPO5	MUCL3	LTBP1	
ZNF384	AQP4	FZD6	
DHX35	SULT1C2	NUAK1	
COQ3	TOR4A	FOLH1	
ZNF639	ALOX5AP	SERPINB13	
USP39	CACNA2D2	STK32B	
RFC2	TMPRSS6	C14orf132	
WDR53	GKN2	TMEM158	
DHX33	ADGRE5	EGR3	
CDKN3	TMEM173	PKP1	
SHCBP1	SERPINB1	SNCA	
ILF3	TESC	MSRB3	
RPAP3	CLDN2	PIGX	
WDR75	C7	TPRG1	
EIF4G1	S1PR4	IGFBP5	
PNPT1	CA13	SIAH2	
SMC6	ME3	FZD1	
MYO19	MAMDC2	PTPN13	
MRPL47	GRAMD2A	DSG3	
POP1	CASS4	TFRC	
H2AFX	AOC3	LRP4	

TUBG1	ALDH2	EFS	
DIP2B	C4B	TRIB2	
CCDC86	SHISA3	ACSS1	
HNRNPR	DUSP1	CNTN1	
UBE2C	CLEC3B	DYNC1I1	
U2AF2	CSDC2	SCN4B	
WDR5	SPTLC3	GNAZ	
HMGB2	OCLN	MB21D2	
CCDC34	KCNN4	CD200	
UPF3B	TENT5C	EPHB3	
NUP153	ABCA8	DDR2	
LTV1	LRRK2	NKAIN2	
TUBA1C	ANKDD1B	WASF1	
PRPF4	C1orf210	HR	
DNAJC14	CA3	PLA2G4F	
SELENOI	ICAM2	SLC37A2	
RBM28	TNFSF13	KRT5	
CCDC138	ENPP5	DLX6	
DDX39A	ATAD3C	KRT6B	
TUBB	VAMP5	CYB5R2	
CENPL	PON2	TIAM1	
RSRC1	TLR2	NPR3	
FAAP24	CABCOCO1	PLCXD2	
TAF5	RGL3	ZNF750	
DEK	KLRB1	LRP12	
IGF2BP3	DNAH5	DSC3	
UBA2	CSRNP1	KCTD1	
TFRC	CREB3L1	GABRQ	
PRKDC	TREM2	LARP6	
ARHGEF39	HLA-DMB	CERS3	
KIF24	CD1A	GPRC5B	
DHX37	SFTPA1	ASAP2	
PGAM5	CXCL2	GPRC5D	
RIOK1	CD52	PDPN	
C18orf54	C11orf96	DNASE1L3	
INTS13	FMN1	TMPRSS11D	
SENP2	RRAD	PARD6G	
RMND5A	ACSM3	SLC1A4	
BLMH	LY96	MTCL1	
FKBP4	CRACR2B	ENTPD3	
XXYLT1	GPSM3	CCND2	
NAA50	EFCC1	CLSTN2	
GPI	SLC41A2	TMPRSS11A	
DVL2	ENC1	IGSF11	

TBCCD1	BANK1	ANTXR1	
KPNB1	RASL12	B3GNT5	
TESMIN	DPYD	KIAA1549L	
NUP155	SLC1A1	SOX21	
NUDT1	C4A	FAM110C	
PTTG1	MICAL2	WIPF3	
DLX6	FAM189A2	CHRM3	
CKS1B	HSD17B6	LPAR3	
NSD2	C5orf49	SOST	
IQGAP3	TLR3	JAKMIP3	
GTF2H3	KCNA3	MTSS1	
PCLAF	TMEM130	B4GALT4	
PATL1	CSF2RA	SCN9A	
FANCE	LRRC31	NDRG2	
FANCC	HPN	DPYSL3	
GFM1	PYROXD2	IGFBP2	
GTF3C5	SGCA	SLIT3	
DDX55	RGS5	EPHA4	
NABP2	WFDC10B	MPPED1	
TOMM40	SNTN	LMO4	
STMN1	GPRC5C	ROR2	
FBL	MS4A2	CCDC3	
KIF20B	CD207	KRT6C	
ABCF3	MUC3A	GRHL3	
RAD51C	OGN	NKD2	
TCP1	SLC43A3	SPRR3	
WASF1	CEACAM4	ATP12A	
SPDL1	MCEMP1	ALDH1A1	
CCT7	CTSS	NUDT10	
TMTC3	FUCA1	DLL1	
NOL11	DAAM2	NECTIN1	
HSPD1	GAS2L2	PRNP	
MPHOSPH9	SFTPA2	ST6GALNAC2	
SENP5	FCGBP	FHL1	
DHX36	XKRX	CYP2S1	
MTHFD2	COLEC12	EPB41L3	
PARP1	C1orf162	SLC22A23	
DNAJC2	PPP1R1B	GOLGA8A	
AFG3L2	VDR	GLYATL2	
TEX10	JCHAIN	WNT3A	
TRIM28	ZBTB16	CYP26A1	
PHGDH	DEPTOR	SPRR2A	
ANAPC1	RHEX	SHISA2	
RNF168	RPH3AL	SNAI2	

CEP85	LPL	SUSD4	
CS	CSF2	ZNF703	
ATP2B1	ZNF385B	HK2	
RANGAP1	FLRT3	IL7R	
TRMT6	SLC6A14	ZNF385A	
TARS	MEOX2	BEX2	
PUS7	EVI2A	STXBP6	
ADCY3	MISP3	BTBD11	
LIG3	CPB2	PTHLH	
KCMF1	FBLN5	FBN2	
TONSL	CAPN9	MLLT11	
LIN9	MFAP4	WNK2	
SIAH2	ERP27	H2AFY2	
CEP76	ERICH2	TNFRSF13C	
YARS2	S100A6	FOXE1	
RCCD1	TCIM	PDGFRA	
NONO	NCMAP	BNC1	
AMER1	ADAMTSL2	BEX4	
PSME3	RASGRF1	SEPTIN5	
TTL	PRR29	LY6G6C	
ACAT2	ABCA3	FAM171A1	
MAZ	NRGN	PLA2G3	
PAK2	BMP5	PLCH2	
STRAP	C6	COL7A1	
SPATS2	PODN	SLC1A3	
PSAT1	RAB37	RARB	
CMAS	C16orf54	CD109	
CHERP	CLIC2	DIRAS2	
NUP62	MS4A15	SPRR1A	
HMGCS1	HHLA2	ACKR3	
HAUS6	VSIG1	KRT13	
SAPCD2	NCF4	USH1G	
ESF1	ALOX5	E2F7	
FOXRED2	CCL23	NELL1	
VTA1	ITGA3	S1PR5	
UBXN7	FABP3	RAB3B	
CENPK	ZMAT1	CECR2	
HMCES	TNFAIP8L2	NPNT	
SUZ12	PCDH1	NID1	
BRD4	SEMA3B	PNCK	
PIGX	TMEM100	C10orf99	
TRMT61B	CPAMD8	SCUBE2	
FIGNL1	ADGRF1	WFDC5	
QTRT2	TENT5A	FAM171B	

HAUS5<br>UBE2O<br>NCBP2<br>SPAST<br>EIF2B5<br>C15orf41<br>OTX1<br>SUV39H2<br>NCL<br>DNMT3B<br>CMSS1<br>C5orf34<br>SYNCRIP<br>POFUT1<br>ANP32B<br>SAP130<br>GTF3C3<br>SBNO1<br>LYAR<br>E2F6<br>POLE2<br>THOC3<br>PDSS1<br>KLHL42<br>QSOX2<br>LRRC58<br>KPNA4<br>POC1A<br>TNPO2<br>CDK16<br>KPNA1<br>PSMD3<br>PAICS<br>ARL6IP6<br>CCDC58<br>CCNE1<br>SAAL1<br>ATP13A3<br>NAA25<br>CDH24<br>CDK4<br>FOXK2<br>SUDS3<br>KNOP1	CLIC6<br>MROH6<br>RASSF7<br>SLC16A7<br>LMO7<br>DEPP1<br>LMOD1<br>SDC4<br>HSH2D<br>IRX5<br>PRSS8<br>FNIP2<br>GLB1L2<br>PLAAT4<br>FRZB<br>AQP5<br>COX4I2<br>MYBPH<br>RASEF<br>ELN<br>ATP13A4<br>DUSP6<br>RND1<br>C9orf135<br>CCR2<br>PID1<br>PDK4<br>C19orf33<br>EVI2B<br>ERN2<br>PRG4<br>CD164L2<br>CACNA1D<br>CFTR<br>PTPN22<br>GOLGA7B<br>UNC5CL<br>OTULINL<br>MILR1<br>PTPRU<br>ICA1<br>RNASE6<br>TM4SF4<br>GAL3ST1	GDA<br>FST<br>AOAH<br>HBEGF<br>FRRS1<br>CSTA<br>IRX6<br>IQCA1<br>CD9<br>HMGCS1<br>FGFBP2<br>FAM83B<br>P3H2<br>NGFR<br>FBXO27<br>SOCS2<br>NUDT11<br>SLC9A2<br>EGR1<br>MSI1<br>ARHGAP23<br>TGM1<br>TGM3<br>SLC6A8<br>SLC47A1<br>PRSS12<br>ARTN<br>SERPINE2<br>ZNF711<br>GPC1<br>SDC2<br>RAB6B<br>ADAM12<br>A2ML1<br>SPRR2E<br>DACT2<br>TRIM29<br>LAMA1<br>PGLYRP3<br>ROBO1<br>HLF<br>HSPB3<br>TPPP<br>SPRR1B	

HMGXB4	FOXJ1	NSG1	
TCOF1	PRDM16	GSTA4	
NELFCD	FUT3	ATP1B3	
CENPP	PPP1R14D	POMC	
POLD3	TFPI	NEFL	
RAN	PTGER2	CES1	
NCBP1	GPR34	FOXN1	
ZMYND19	SLC6A20	C12orf75	
SLC38A1	GPD1	MXRA5	
FGD1	MRC1	PLPPR1	
ZC3H8	BICDL2	HSPG2	
ALG3	TSPAN8	KCNJ15	
MFN1	CLDN3	ADRA2B	
TSR1	C4BPB	FOXD1	
ACAP2	ZNF626	LOXL4	
FAM136A	FAM216B	CDK6	
APEX2	NT5E	GJB6	
TPRKB	EDNRB	FAM83F	
ERAL1	FOLR2	SULF2	
POLD1	GPIHBP1	NPPC	
CCT5	HLA-DOA	ARNT2	
CARM1	ZMYND10	SPOCK1	
SLC5A6	PIP5KL1	FMO2	
MARK1	RSPH1	POU2AF1	
EIF4A3	IYD	KRT6A	
IPO9	KBTBD11	SPRR2D	
JARID2	PLN	IGF2BP2	
WNK2	PIP	AADAC	
CASP2	REEP6	EFNB1	
KDM1A	IRX3	FZD10	
NUP93	BATF	CDH26	
FIP1L1	CLTRN	COLCA2	
CHTF18	TNXB	FABP5	
SUPT16H	PLEKHB1	DSC2	
PIK3R4	SERPIND1	PGF	
MLF2	HOXD1	CEL	
URB2	COL8A1	KRT15	
SOGA1	TOX3	SV2A	
VRK2	ARSD	CCNA1	
NDUFAF4	ENPP3	COL6A3	
FYTTD1	DDIT4L	C6orf15	
DDX11	PHYHD1	GPR27	
PLAGL2	DES	THBD	
STARD7	SLC22A18AS	EN1	

NCBP2AS2	HHIP	PENK	
PUS1	BAAT	SLC4A11	
GANAB	HLA-DQA1	CAPNS2	
PDAP1	GJA5	EYA2	
CENPJ	TLE2	SLC9A3R1	
NEDD1	MMRN1	RND3	
ZGRF1	RTN4RL2	TNFRSF19	
KDM3A	GDF10	GAS1	
PTDSS1	NMNAT2	CHRDL1	
SNRPD3	NPR1	GAST	
SF3B3	TGM2	HCAR2	
SOX2	CCL17	MFAP4	
RPF2	DAW1	MSC	
POLR2H	ECRG4	GABRA3	
SMARCB1	DTX4	RAPGEFL1	
HMMR	CADPS2	CALB2	
U2SURP	WFDC3	IRF6	
CLCN2	CLDN18	NTS	
LRPPRC	PLCH1	WDR72	
FAM111B	TRPC6	NDUFA4L2	
NOP58	LIPH	SPRR2G	
EXOSC3	SSC5D	TNFSF10	
ECE2	PTGDS	ADM	
MAP6D1	TMED6	SPON1	
LCLAT1	TBX4	SELENOP	
CSRP2	PODNL1	SCG2	
DONSON	FAM177B	ITGA2	
TOP3A	SPATA18	HOXD11	
RAD23A	VWA2	FAM83C	
SLC16A1	ADRA2A	NXPH4	
ATXN2L	COBL	DIO2	
TMEM189	C2	KCNG1	
UBAC1	C7orf57	TMEM40	
GAPDH	MMP15	HOXD13	
ACAD9	IGSF6	SPINK5	
DBR1	EFNA5	ALDH3A1	
SNRNP200	ITM2A	TINCR	
HDHD5	CCL13	HOXD10	
RBBP7	CSF3R	NRARP	
ELK1	OLFML3	KLRG2	
PES1	BAIAP3	CA12	
B3GNT5	MISP	CPA3	
ANKRD52	OLR1	PERP	
DHX34	CD300LF	KRT16	

CBX2	KCTD14	GSTM3	
XRCC3	CLEC10A	B4GALNT1	
PHF6	MS4A8	NTN1	
NAT10	TMC4	AMTN	
CRKL	KPNA7	WIF1	
MRPL3	FCMR	KRTDAP	
CTSV	NR0B2	CYP4F3	
CSTF2	LPIN2	TNC	
SNRPB	KDR	SBSN	
TIMM44	SPNS2	IGSF9	
DDX49	CLDN9	CX3CL1	
CCNT1	CST2	COL14A1	
SMC3	RASD1	RHCG	
CPSF4	NTN4	SERPINB5	
CNOT3	AZGP1	GRP	
LRRC8D	FAM92B	FGFR3	
SAMD4B	CIITA	CHP2	
ABCC5	CADM1	IL36G	
MTERF3	C20orf85	SOHLH1	
HDAC1	MNDA	GJB5	
TPI1	CGN	KRT31	
PRPSAP1	CGNL1	IVL	
KRI1	DMBT1	LAMA3	
RAD21	PLEKHA6	ARHGEF4	
CCDC150	TNFSF15	KRT17	
ARTN	CAPS	CYP2C18	
UBXN2A	FCN1	FMO3	
TDP1	CAPN5	TPD52L1	
GAREM2	SLPI	LGR5	
TMEM237	COMP	IL1RN	
TWNK	TUBA4B	COL17A1	
NUDT15	ABI3BP	SLC16A1	
CEP97	FBXO2	CXCL14	
HCFC1	RAB27B	SERPINB2	
ZW10	GEM	POF1B	
CSNK2A1	GPR183	ANXA8	
RMI1	EPHX3	PI3	
TFAP4	CD69	DQX1	
PNO1	ERRFI1	PPP2R2C	
NUP188	AMIGO2	RBP1	
SMARCD2	VEGFD	MAGEA11	
CCNE2	FOS	XG	
DCUN1D1	MST1R	SIX2	
ALG10	MACROD2	KRT14	

LSM14A	ACKR1	PHGDH	
TCF3	CYP27A1	RNASE7	
ZNF131	PMEPA1	F13A1	
EHBP1	AOC1	IGFL1	
PPAT	MXRA8	NIPAL4	
MIEF1	SLAIN1	PPP1R14C	
BRIX1	HID1	TSPYL5	
ATP2C1	GALNT5	MMP10	
CCT4	ISM1	VTCN1	
HBS1L	TFPI2	CLDN1	
METTL8	MYO1G	FOSB	
NLE1	ADORA1	UPK1B	
DUS3L	BTC	CRCT1	
ODF2	DNAJC12	SFRP1	
MAGEF1	TYROBP	ULBP2	
C12orf56	TEKT1	CYP4F11	
ADAM17	ST6GALNAC1	SOX15	
TGIF2	HSPB6	COL12A1	
GGH	TSPAN12	UGT1A6	
DLG1	AGT	TMPRSS4	
GPR19	SLCO2A1	C7	
USP1	PLEKHS1	NOS2	
PARL	SFTPC	LGALS7	
PRAME	TCEA3	LGALS7B	
FANCL	MUC5B	SPRR2F	
AGO2	TNFRSF12A	SERPINB3	
NUP50	CRTAC1	GSTA1	
MDH1	ZNF117	SLC2A1	
DDX54	UCN3	MMP3	
ZNF318	PRELP	FGFBP1	
DLX5	IL33	AKR1B10	
HDGFL3	VASN	KRT75	
WIZ	SPON1	SERPINB7	
NEIL3	GDF15	FXYD3	
TAF2	CCL22	LYPD3	
TMEM185B	MAB21L4	AKR1C1	
MAPK6	SH3BGRL2	S100A2	
FARSA	TMEM61	ADH1B	
CCDC137	MSR1	MMP12	
PDCD5	HGD	GPX2	
HPRT1	AGER	PRAME	
PAK1IP1	SLC40A1	SERPINB4	
TBPL1	TMEM119	LTF	
STIP1	MARCO	CALML5	

NASP	TNFRSF17	TNS4	
ATP11B	BMP2	GPR87	
DOT1L	CDC42EP5	S100A7	
PRMT1	PTK6	AKR1C2	
ASAP1	PHACTR3	MAGEA4	
PRICKLE3	SCGB2A1	AKR1C3	
E2F8	C1orf189	SCGB1A1	
TGS1	RAP1GAP		
PFN2	CHRDL1		
IQCB1	CPA3		
BCL7A	LTK		
HAP1	BSPRY		
NRM	CAPN13		
ZC3HAV1L	C1orf194		
GNGT1	BRINP1		
HYLS1	HP		
TIMM8A	ATP2C2		
FBXO43	DPT		
CLPB	MT1E		
MDC1	ASPHD1		
CASP8AP2	PAEP		
ZNF74	ECHDC3		
DNMT3A	TMEM59L		
PEX5	METTL7B		
GSK3B	MORN5		
SMO	HBB		
KIF3C	GADD45G		
ABCC1	PPBP		
PELP1	ZNF486		
STC2	BMP3		
MSI1	PTPRN2		
MRGBP	LGSN		
PSMC4	CAMK2N1		
ALKBH2	AREG		
SLC2A1	PALM3		
PXYLP1	WDR38		
FAM222A	TNNC2		
ZNF777	PCP4		
SAFB	TSPAN1		
GTPBP3	BCAS1		
IPPK	LCN2		
TNPO3	ALPL		
UNC119	FMO2		
RPAP1	SYT12		

ZNF343	GCNT3		
RNF26	VSIG4		
ASNS	SPAG6		
UBAP2	TRNP1		
FAM83F	PLAAT3		
ANKRD13B	C11orf86		
SMIM13	MMP7		
HOXD13	TPSB2		
PARP2	SCGB1A1		
ZNF146	ASPN		
FAM189B	ALPK3		
HSF2	FABP4		
PIK3CA	C9orf24		
NXPH4	CH25H		
IGSF9	RAMP1		
MICAL3	LDLRAD1		
FAM234B	FGG		
DDX10	HBA2		
LYPD6	B3GNT3		
SMG7	BPIFB1		
DIPK2A	MUC5AC		
MTMR1	ACHE		
PGAP1	CRLF1		
CKS2	TPPP3		
RPIA	LGALS4		
COLGALT1	RNF128		
CEP250	AMBP		
SLC6A8	FXYD4		
RDM1	BPIFA2		
B4GALT6	LYZ		
BYSL	FAM83A		
TMEM38B	SFRP4		
AGBL5	CDA		
TFDP1	S100P		
PCYT1A	CP		
EPOP	BPIFA1		
ABCF1	CEACAM5		
YWHAG	EREG		
WDR4	PCP4L1		
SINHCAF	TFF3		
MLLT11	BHMT2		
LSM4	HLA-DQA2		
PPME1	KIT		
KLHL13	COL10A1		

ANKRD27<br>ATG4D<br>PPIF<br>POLD2<br>SLC7A1<br>KLC2<br>POLR1A<br>ZNF507<br>MED30<br>MRPS22<br>PTPN11<br>STK35<br>FAM83B<br>PHF8<br>GCN1<br>AP2M1<br>AHCY<br>GIT1<br>CEP72<br>GABRA3<br>TAF1A<br>POLE3<br>MSL2<br>OXGR1<br>DNAJC19<br>ACLY<br>CHRM3<br>LRP4<br>FAM20B<br>PITPNB<br>DSP<br>NRARP<br>SMIM10L1<br>CBFA2T2<br>TRIM25<br>B3GALNT2<br>CORO1C<br>C20orf27<br>IGF2BP2<br>CD3EAP<br>CDV3<br>PRRC2B<br>LRRC1<br>NCOA6	CHIT1<br>CCL19<br>MMP13<br>FGL1<br>TCN1<br>FGA<br>TFF1<br>CRABP2<br>EEF1A2		

EMC1<br>SFXN1<br>ZNF280C<br>ASAP2<br>DROSHA<br>NDE1<br>RBM19<br>SLC3A2<br>KCTD1<br>PIGW<br>CHAC2<br>QSER1<br>CACNA1B<br>MRPL51<br>NSUN2<br>BOLA3<br>CSNK1G1<br>PDCD10<br>TMEM117<br>TEAD4<br>TCF20<br>APPL2<br>PDRG1<br>POM121<br>RPL39L<br>PREP<br>RAVER1<br>USP31<br>C19orf47<br>ALMS1<br>RECQL<br>IGSF11<br>WDR72<br>NFE2L1<br>METAP1<br>GCLC<br>SMS<br>FAM131C<br>SLC7A5<br>GDAP1<br>RCOR2<br>DARS2<br>CENPQ<br>LBR			

TRIM59<br>IARS<br>TEDC1<br>B4GALT4<br>GEMIN4<br>SEM1<br>HEATR1<br>HOXA1<br>TULP3<br>RBBP8<br>CAND1<br>PFKM<br>CKAP4<br>SAMD1<br>FOXD1<br>ATP2A2<br>HSPA4L<br>PCCB<br>CCSAP<br>TBL1XR1<br>YES1<br>DCUN1D5<br>ZNF488<br>PRKX<br>TTLL12<br>CAMSAP1<br>SV2A<br>ADAM23<br>POP7<br>HIC2<br>POM121C<br>CEP19<br>DHCR7<br>YEATS4<br>ZNF695<br>CCDC85C<br>FBXO27<br>BARD1<br>DHFR<br>CHORDC1<br>SF3A2<br>PPT2<br>FAM210A<br>TMEM14A			

NHSL1<br>SH3BP1<br>ATR<br>ATP1B3<br>LARP6<br>MARS2<br>CDK5R1<br>COL7A1<br>STAG1<br>MTCL1<br>NADK2<br>AEBP2<br>VPS8<br>CASTOR2<br>LRR1<br>TIMMDC1<br>HSPE1<br>ARID3B<br>PHF5A<br>BCL2L12<br>FOXE1<br>EIF4EBP1<br>PCDH19<br>CCT6A<br>DPF1<br>KCTD3<br>PIR<br>RCN2<br>TOMM5<br>SIM2<br>ELOVL4<br>TMEM44<br>TENT4A<br>MPP2<br>LSM14B<br>DMRT2<br>DTYMK<br>PKP4<br>NRCAM<br>PRMT5<br>LRP8<br>KRT74<br>FGF12<br>OTUD6B			

ATP23<br>FAM169A<br>AP3M2<br>PHLPP1<br>ZNF385A<br>YBX1<br>DLG5<br>KIF21A<br>PLD1<br>COMMD2<br>NAB1<br>WDR18<br>USP28<br>C19orf54<br>SOX21<br>DYNC1I1<br>ZBTB5<br>REXO5<br>HSP90AA1<br>CEP192<br>RNF126<br>HOXD11<br>SLC25A33<br>ACYP1<br>ICK<br>GABRQ<br>MEST<br>LIPT2<br>MELTF<br>GPR63<br>NTRK2<br>DRG1<br>CNTNAP2<br>C3orf67<br>LRP12<br>APOBEC3B<br>SLCO1A2<br>B4GALNT1<br>NKAIN2<br>YDJC<br>DHRS13<br>PHB2<br>DDIT3<br>ANKRD18B			

WDR76<br>APOO<br>GNA13<br>MTHFD1L<br>PNCK<br>CPOX<br>ADAMTS20<br>CSNK1G2<br>SERPINB5<br>ECSIT<br>CLTCL1<br>SNRPG<br>TFDP2<br>RFX7<br>VANGL2<br>TRMT10C<br>NIPSNAP1<br>ASIC1<br>RAB3B<br>GLTP<br>NECTIN1<br>SKA2<br>CDCA7<br>KIF7<br>TMEM41A<br>TWF1<br>LRFN1<br>PAK1<br>HOXA10<br>CLUH<br>FAM162A<br>E2F3<br>CLASP1<br>GOLIM4<br>ADSL<br>SARS2<br>MBD3<br>BOP1<br>S1PR5<br>DSC3<br>SUGP2<br>M6PR<br>MICALL1<br>KEAP1			

DCAKD<br>PDCD11<br>DUSP14<br>PGM2<br>B4GALNT4<br>BICD2<br>MYBBP1A<br>ZNF770<br>ERF<br>MIB1<br>NSD3<br>CCT2<br>SRSF12<br>MTA1<br>GNL3L<br>HOXC8<br>HK2<br>KCNG1<br>NT5DC3<br>MYNN<br>TAF4B<br>EIF5A2<br>H1FX<br>ADAM11<br>GPSM2<br>STOML2<br>PAK4<br>AMMECR1<br>TRIM45<br>MAFG<br>FAM83G<br>HRAS<br>ZIC5<br>ZNF711<br>STRBP<br>URI1<br>PERP<br>NMD3<br>DTNB<br>REEP4<br>ABRACL<br>ICE1<br>FOXD3<br>GPR161			

SLC6A15<br>GRHL2<br>SLC35G1<br>CHST14<br>DLX1<br>ZBTB12<br>FRRS1<br>SHOX2<br>LHX2<br>GNB1L<br>NR2C2AP<br>RNF7<br>TMEM132A<br>RPP40<br>PM20D2<br>MORC4<br>PRR14L<br>PROSER3<br>BMP7<br>PDK1<br>DSG3<br>DLK2<br>PRELID3A<br>PTMS<br>FBN2<br>ZKSCAN1<br>PLEKHG6<br>EMILIN3<br>DDX21<br>SLC9A3R1<br>TRIM16L<br>CDCA7L<br>RYK<br>USH1G<br>OTULIN<br>TP63<br>FAM161A<br>EHHADH<br>LDLRAD3<br>DMRT3<br>TMEM161A<br>CCDC51<br>NMNAT3<br>POLR2A			

MRPL4<br>CABLES2<br>TMLHE<br>ZNF598<br>RHBDD3<br>NDRG4<br>PAX6<br>KIAA1549<br>FBXL19<br>JUP<br>SLC39A6<br>IGF2BP1<br>BDH1<br>SYT14<br>ADM<br>DSG2<br>CALML3<br>SEMA3F<br>TFAP2A<br>FSD1<br>EPHA7<br>HS6ST1<br>CRYBB3<br>FAM117B<br>PCYT1B<br>ERMP1<br>NKAIN1<br>ULK1<br>ISL2<br>PARD3<br>FBXL14<br>PRRC2A<br>PRDM13<br>ZIC2<br>ADD2<br>ZNF703<br>ITGA6<br>BCL11A<br>KMT2B<br>CELSR2<br>CERS6<br>GPC2<br>TUFT1<br>HSPH1			

LLGL1<br>TMEM106C<br>ARNTL2<br>KHDC1<br>CABYR<br>HIST1H2BH<br>SQLE<br>ADA<br>CLDND1<br>FAM131A<br>SLC1A5<br>TRPV4<br>WASF2<br>PNN<br>VDAC3<br>RAG1<br>CORO6<br>STK38L<br>SOX12<br>RAPGEFL1<br>HECA<br>DAPL1<br>SLC25A36<br>COCH<br>PTHLH<br>MAP3K4<br>EIF4A2<br>PRKCI<br>KRT5<br>RIMS2<br>FZD6<br>CDK6<br>BOLA2-<br>SMG1P6<br>TCTEX1D2<br>SREBF2<br>MCCC1<br>CNIH2<br>EFS<br>HAUS1<br>EXOC6B<br>SOWAHC<br>ACOT7<br>FBXO41			

TRIM16<br>P2RY1<br>RHEBL1<br>NUDT11<br>MZT1<br>KREMEN2<br>ADRA2B<br>MRAP2<br>PGD<br>C1QBP<br>CASK<br>GABRR1<br>JAG1<br>PARD6G<br>NPM3<br>PPP2R3B<br>COL4A6<br>H2AFY2<br>EDARADD<br>C21orf58<br>HOXA13<br>KLRG2<br>KCTD15<br>WEE1<br>ATN1<br>DLL1<br>MKRN3<br>NDUFB5<br>PGAM1<br>HLTF<br>PSPH<br>USP46<br>ATP6V1E2<br>RRS1<br>NTS<br>HOXC6<br>GBP6<br>TMEM158<br>CHCHD6<br>TESK2<br>PHLDB3<br>ELF4<br>ZNF280A<br>ABHD3			

ZFYVE9<br>NFE2L2<br>ULBP1<br>RAET1L<br>YBX3<br>ACP7<br>HPDL<br>MAP7D3<br>SOX15<br>LRFN4<br>MYB<br>HOXD10<br>RNF217<br>ARHGEF5<br>BTBD11<br>ISM2<br>PTPRS<br>DSC2<br>RPP25<br>MRPS12<br>CNNM1<br>NCR3LG1<br>DUSP9<br>MAPK12<br>HEY1<br>TRRAP<br>MAPKBP1<br>HSPA1B<br>PRDM11<br>PLEKHG3<br>RELL2<br>CLEC2L<br>ERVMER34-1<br>UCHL1<br>SIX2<br>HOXA11<br>ACVR1C<br>RAVER2<br>FSCN1<br>NKX2-5<br>PFKFB4<br>HMGA2<br>NUMBL<br>RARG			

PRTFDC1<br>ZNF724<br>TBL1X<br>PPFIA3<br>LRATD1<br>GRHL3<br>TMEM17<br>TCF15<br>STK26<br>PDK3<br>FOXL2<br>NXPE3<br>FAT2<br>PTGFRN<br>SOCS7<br>SERTAD2<br>SCARB1<br>TPD52L1<br>GAST<br>MARCKS<br>STON2<br>CYC1<br>HILPDA<br>CYB5R2<br>TRIM29<br>IRF6<br>LMO4<br>PKP1<br>HTR2C<br>DQX1<br>CLSTN1<br>TNK2<br>PKP2<br>VSNL1<br>FRRS1L<br>GDF11<br>NCK1<br>NT5M<br>PTPRZ1<br>TMEM246<br>SLC38A2<br>FZD10<br>BNC1<br>PLXNA1			

ALDOC<br>ARHGEF4<br>DST<br>FAM43A<br>IL22RA1<br>ADH7<br>PSIP1<br>RIPPLY3<br>ME1<br>A4GALT<br>FST<br>TINCR<br>SEPTIN5<br>POMC<br>NPPC<br>TMEM97<br>KRT6A<br>COLEC11<br>CYP4F3<br>DNAJB1<br>COL4A5<br>RASSF9<br>POPDC3<br>TRIB3<br>NCS1<br>PITX1<br>FJX1<br>KRT13<br>FOXK1<br>UCN2<br>TMTC1<br>CEL<br>ATRN<br>CHRNB4<br>FOLH1<br>POMK<br>XCL1<br>PRKG2<br>PTPRF<br>SYT1<br>MYC<br>IGF1R<br>SLC4A11<br>DDR1			

C21orf91<br>SLC2A12<br>WDR54<br>ALX1<br>CERS3<br>SLC9A2<br>ZNF280B<br>LYPD6B<br>AP3B2<br>DGKA<br>FADS2<br>OXCT1<br>BID<br>SLC5A12<br>KRT15<br>SMOX<br>CSTA<br>TSPAN33<br>GJB7<br>FAM171B<br>COLGALT2<br>FRAS1<br>WIPF3<br>NETO2<br>SORD<br>PHLDB2<br>G6PD<br>UGT1A6<br>TSKU<br>PAX9<br>TMEM40<br>GJB5<br>CHST7<br>FAM181B<br>BEX3<br>DZIP1<br>STAR<br>CIB2<br>ETNK2<br>SNAI2<br>LAMA1<br>CDC25B<br>SH2D5<br>MAGEA11			

SLC51A<br>UAP1L1<br>SEMA6A<br>KLHL24<br>RND3<br>PTH2R<br>SLC12A8<br>GCLM<br>PAK5<br>GJA3<br>ACKR3<br>CHODL<br>ATF5<br>CNTN1<br>KIAA1671<br>GPR156<br>FZD7<br>PPFIA4<br>EEPD1<br>TPRG1<br>STOX2<br>SRXN1<br>SYNGR1<br>PPP2R2C<br>FAM83C<br>FOXN1<br>RNF165<br>UGT1A7<br>GDA<br>DGKG<br>INHBE<br>NUP210<br>MYCL<br>CLGN<br>SCD<br>PRMT6<br>LIMK2<br>RGMA<br>BAG4<br>GNG4<br>CDK5RAP2<br>CACNA2D3<br>PPP1R3G<br>LYPD3			

RND2<br>GPC1<br>PLEKHG4<br>HOXB13<br>NDUFA4L2<br>EYA2<br>PLEKHG5<br>DCAF12L2<br>ELOVL6<br>NEURL1<br>SERPINB13<br>KRT6B<br>SEMA6D<br>PRIMA1<br>KRT31<br>FRMD6<br>NECAB2<br>FOXL2NB<br>DENND2C<br>C12orf75<br>ELOVL2<br>MID1<br>INA<br>AK4<br>CHRNA5<br>CD9<br>TP73<br>CD109<br>PNPLA3<br>KRT17<br>OGDHL<br>ADGRL3<br>KLHL5<br>RBM38<br>MCOLN3<br>TMPRSS11F<br>GPRC5D<br>RAP2B<br>C1QL1<br>UPK1B<br>SHROOM2<br>ANKRD2<br>ALOX12<br>SPHK1			

GNAZ<br>CLCA2<br>BICDL1<br>CHAC1<br>FADS1<br>SIX4<br>PRDX1<br>P3H4<br>PMAIP1<br>VAX2<br>IGSF3<br>CDHR1<br>SNX31<br>MAP1B<br>LTB4R<br>HIST1H3B<br>HOXA9<br>NRN1<br>CKMT1B<br>SLC20A2<br>ALDH3B2<br>RAB6B<br>MPPED1<br>JAG2<br>ALG1L<br>ARHGEF25<br>PHF24<br>CACNA2D1<br>KLC3<br>FGFBP1<br>HIST1H2BJ<br>RHCG<br>GSTA4<br>TBX1<br>KIAA1549L<br>B3GALNT1<br>NOTCH1<br>CCL26<br>HOXB7<br>TRO<br>GPRC5B<br>DNAJB4<br>TMSB15A<br>GJC1			

BIK<br>F12<br>MAMSTR<br>KCNK2<br>HR<br>LPAR3<br>LAMA5<br>PLEKHH1<br>SLC1A4<br>TMPRSS11A<br>CENPV<br>CBX6<br>SERPINE2<br>IGFBP2<br>SOHLH1<br>SDK1<br>ABCB6<br>SLC39A14<br>A2ML1<br>TDRD5<br>ST6GALNAC2<br>LEF1<br>SLC7A11<br>NEO1<br>AJUBA<br>CYP26A1<br>FERMT1<br>TBX18<br>DNM1<br>HOXC9<br>TENM2<br>JAKMIP3<br>HOXD9<br>SPTBN2<br>CSAG3<br>MAGEA4<br>LHX5<br>AKR1B10<br>ASCL2<br>MMP12<br>ARHGAP33<br>ZP3<br>PPM1L<br>FBXO17			

NOTCH3<br>KRT16<br>CAMK2N2<br>FGFR2<br>HIST1H2AG<br>MIF<br>KLF4<br>DPYSL4<br>TXNRD1<br>TMEM171<br>NRG1<br>SNCA<br>MAGEA3<br>ULBP3<br>EPHB3<br>MAGEA6<br>ABCA13<br>TUBB4A<br>POU6F2<br>PKP3<br>KRT33A<br>WNT3A<br>NELL2<br>KIF1A<br>EN1<br>GPX2<br>RTN4R<br>CYP4F11<br>DMRTA2<br>TMEM79<br>SPRR2A<br>KLF5<br>HIST1H2BO<br>IL23A<br>GPRIN1<br>UGT8<br>GAL<br>ADGRB2<br>EPN3<br>ODC1<br>PLA2R1<br>XG<br>EDDM13<br>KRT77			

PCSK9<br>LTB4R2<br>NAV1<br>CLDN20<br>TNS4<br>TUSC3<br>LY6G6C<br>MERTK<br>CKMT1A<br>SPRR1A<br>MB21D2<br>ZIC1<br>GRHL1<br>C16orf74<br>NOS2<br>ANXA8<br>SDK2<br>ARHGEF26<br>RAC3<br>NTN1<br>CYP2S1<br>ADGRL1<br>MATN2<br>STYK1<br>ULBP2<br>GAP43<br>FETUB<br>WNT2B<br>SGK1<br>YJEFN3<br>ARHGEF19<br>MLF1<br>SERPINB2<br>TXN<br>KRT6C<br>GPNMB<br>ARL4D<br>CAPNS2<br>TRIM7<br>CALB1<br>BCL2L10<br>ARHGAP23<br>SOST<br>CADM4			

IRX4<br>AMOTL1<br>HOXA3<br>EPHB1<br>MAPK8IP2<br>RPL22L1<br>CSAG2<br>TUB<br>DIRAS2<br>HOXC13<br>RIPPLY2<br>CIART<br>GJB3<br>FAM171A2<br>RPRM<br>GLI2<br>C10orf99<br>SIPA1L2<br>SERPINB12<br>PLAAT1<br>KRT14<br>HOXD8<br>GPR50<br>EPHA1<br>GALNT13<br>GJB6<br>PI3<br>PLOD2<br>ITGB8<br>COLCA2<br>MDGA1<br>GRIN2D<br>ALDH1A1<br>HCAR3<br>GSTM3<br>GPC3<br>PLA2G3<br>PTGR1<br>GSTM4<br>MCC<br>DSG1<br>STEAP1B<br>EFNB1<br>TFAP2C			

JAKMIP2			
KCNJ2			
CITED4			
VWDE			
BCL2			
SPRR3			
SLCO3A1			
SCD5			
FOSL1			
SOSTDC1			
GSR			
WNT5A			
SLC7A8			
PLAC1			
SEMA3D			
C6orf15			
EPHB6			
CYP2W1			
SIX3			
OSR2			
PLCB4			
TUBB2B			
NUDT10			
TUBB6			
FGFBP2			
TMPRSS11D			
PLCXD2			
C12orf54			
MAGEA1			
KRT19			
ROBO1			
SPOCK1			
HOXB9			
CLDN1			
RUNX3			
SEPTIN3			
HIST1H2AE			
ALDH3A1			
PADI3			
RHOV			
HCAR2			
AKR1C2			
GJB4			
NDRG1			

GPR87<br>QRFPR<br>SPRR2E<br>RBP1<br>SFN<br>BCL11B<br>STC1<br>NAP1L2<br>CALML5<br>UST<br>IFI16<br>PGF<br>LRRC4<br>NEFL<br>JPH1<br>LGALS7<br>LGALS7B<br>CBLC<br>BEX2<br>FAM171A1<br>GSTM2<br>PGBD5<br>MEX3A<br>MT1X<br>PANX2<br>RPTN<br>MN1<br>MAGEB2<br>SLC4A3<br>HSPB1<br>POF1B<br>CCDC190<br>TGM1<br>KRT78<br>SULT4A1<br>SLC39A2<br>OVOL1<br>YBX2<br>CDKN2A<br>NPBWR1<br>GJA1<br>BEX1<br>GHR<br>CSAG1			

AKR1C1<br>F2RL2<br>DLL3<br>COL9A3<br>ANXA8L1<br>S100A2<br>CNFN<br>MAGEA10<br>TNFRSF18<br>KRT75<br>FOXI3<br>TGM3<br>LAMA3<br>OSGIN1<br>CGREF1<br>WFDC5<br>NGFR<br>FABP5<br>HES2<br>DKK1<br>GLYATL2<br>GPR27<br>HOXB8<br>FAM110C<br>HOXC10<br>TSPAN18<br>CA12<br>NPTXR<br>IGFBP5<br>CRYBG2<br>NR0B1<br>CBR3<br>CCNA1<br>FHOD3<br>SBSN<br>SPRR1B<br>MAGEA12<br>IL1R2<br>SPRR2G<br>SPRR2D<br>BARX1<br>IL20RB<br>TWIST1<br>HIST1H2BG			

PGLYRP3<br>PTN<br>SULF2<br>EGLN3<br>ADAM12<br>CHST2<br>SERPINB3<br>TP53AIP1<br>KRTDAP<br>CRABP1<br>CHP2<br>PLCH2<br>ATP12A<br>C1QTNF12<br>LGR5<br>NSG1<br>PRSS21<br>COL17A1<br>WNT11<br>SESN3<br>SERPINB7<br>ITGB4<br>GABRE<br>TSPYL5<br>FXYD3<br>SPTSSB<br>LY6K<br>DACT2<br>HIST3H2A<br>SFRP1<br>AMTN<br>FGFR3<br>MMP10<br>CALB2<br>ZBTB7C<br>PRR15<br>AKR1B15<br>SPRR2F<br>RNASE7<br>HAS3<br>IGFL1<br>GSDMC<br>ABCC2<br>AKR1C3			

GJB2<br>GSTA1<br>IL36RN<br>IL1RN<br>SERPINB4<br>VTCN1<br>CDH3<br>CHGB<br>CLCA4<br>TMPRSS11E<br>IL1A<br>PEG10<br>S100A7<br>TNNT1<br>CA9<br>DDX3Y<br>IL36G<br>KLK10<br>CES1<br>RPS4Y1<br>S100A8			
